# High strength and damage-tolerance in echinoderm stereom as a natural bicontinuous ceramic cellular solid

**DOI:** 10.1038/s41467-022-33712-z

**Published:** 2022-10-14

**Authors:** Ting Yang, Zian Jia, Ziling Wu, Hongshun Chen, Zhifei Deng, Liuni Chen, Yunhui Zhu, Ling Li

**Affiliations:** 1 Department of Mechanical Engineering, Virginia Tech, Blacksburg, VA 24061 USA; 2 Department of Electrical and Computer Engineering, Virginia Tech, Blacksburg, VA 24061 USA

**Keywords:** Mechanical properties, Bioinspired materials

## Abstract

Due to their low damage tolerance, engineering ceramic foams are often limited to non-structural usages. In this work, we report that stereom, a bioceramic cellular solid (relative density, 0.2–0.4) commonly found in the mineralized skeletal elements of echinoderms (e.g., sea urchin spines), achieves simultaneous high relative strength which approaches the Suquet bound and remarkable energy absorption capability (ca. 17.7 kJ kg^−1^) through its unique bicontinuous open-cell foam-like microstructure. The high strength is due to the ultra-low stress concentrations within the stereom during loading, resulted from their defect-free cellular morphologies with near-constant surface mean curvatures and negative Gaussian curvatures. Furthermore, the combination of bending-induced microfracture of branches and subsequent local jamming of fractured fragments facilitated by small throat openings in stereom leads to the progressive formation and growth of damage bands with significant microscopic densification of fragments, and consequently, contributes to stereom’s exceptionally high damage tolerance.

## Abstract (web summary)

Engineering ceramic foams are often limited for non-structural usages due to their brittleness. Here the authors elucidate the structural design strategies of echinoderm stereo as a biological ceramic cellular solid for achieving simultaneous high strength and damage tolerance.

## Introduction

Biological structural materials in organisms’ exo- and endoskeletons often rely on dense composites consisting of minerals and organic materials organized in a complex, hierarchical manner, which allows for efficient strengthening and toughening mechanisms^1–3^. Oftentimes organisms also require weight reduction in their skeletal systems due to other functional requirements, such as body support and locomotion, as well as minimization of energy costs^4^. By introducing porosity, cellular solids represent an effective solution and are widely exploited in nature with a diverse variety of cellular designs, such as wood, trabecular bone, and bird feather^4, 5^. While these structures are often based on organic or composite materials, fully mineralized cellular solids in nature are considerably rare^5^. One of the fundamental challenges of using minerals or ceramics for constructing cellular solids is their brittleness, which often results in catastrophic failure instead of elastic buckling or plastic yielding as in soft or ductile materials^6^. Despite recent progress in using 3D printing to fabricate ceramic lattices^6–10^, most synthetic ceramic cellular solids exhibit low damage tolerance and are often limited to non-structural applications, such as catalyst support, filtering, burning media, etc^11^.

In this work, we investigate the material design strategies of the biomineralized, porous sea urchin spines as a biological ceramic cellular solid for achieving high strength and improved damage tolerance, which can provide important insights for the development of advanced ceramic cellular solids. Quantitative 3D structural characterization coupled with finite element analysis demonstrates that, despite being a bending-dominated structure with a low average nodal connectivity of 3.3, the porous stereom structure of sea urchin (*Heterocentrotus mamillatus*) spines (relative density, $\bar{\rho }$ 0.2–0.4) achieves high strength due to low stress concentrations resulted from the unique smooth branch and node morphology, which exhibits near-constant surface mean curvatures and negative Gaussian curvatures. Combined synchrotron-based in situ mechanical characterizations, electron microscopic analysis, and computational modeling further reveal that upon mechanical loading, the porous stereom structure undergoes graceful failure through the formation of densified damage bands. During this process, the fractured branches are efficiently jammed by the small throat openings (diameter, ~20 μm) within the bicontinuous cellular structure, which contributes to the formation of damage bands with densely packed microscopic fracture fragments. The continuous widening of the damage bands through progressive microfracture of branches contributes to the high energy absorption and damage tolerance of this unique natural ceramic cellular solid.

## Results

**Fig. 1:**
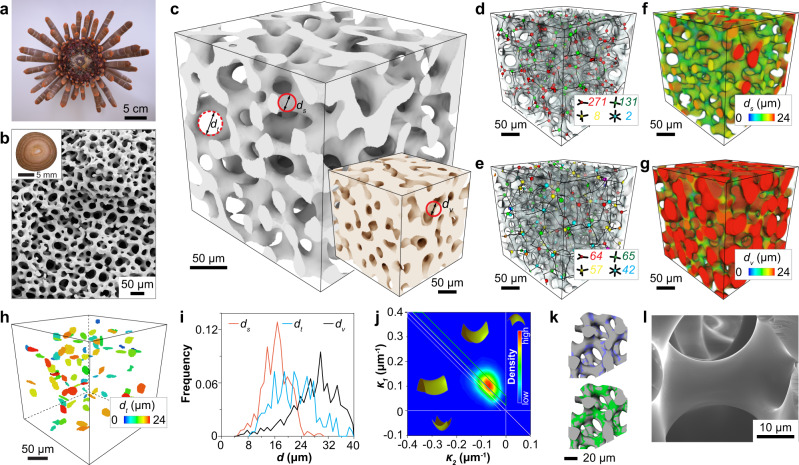
Echinoderm stereom as a bicontinuous cellular solid. **a** Photograph of a ventral-viewed sea urchin *H. mamillatus*. **b** SEM image of the stereom structure. Inset, optical image of the transverse cross section of a spine. **c** µ-CT reconstructions of stereom and the corresponding void structure (inset). *d*_*s*_, *d*_*v*_, and *d*_*t*_ represent the thicknesses (diameters) of stereom, void structure, and throats, respectively. **d**, **e** 3D cellular network of stereom and the corresponding void structure with node types colored by their connectivities. **f**, **g** The thickness distributions of stereom (*d*_*s*_) and the corresponding void structure (*d*_*v*_). **h** 3D rendering of small throats (*d*_*t*_ < 24 μm) for volume (**c**). **i** Distribution of *d*_*s*_, *d*_*v*_, and *d*_*t*_. **j** Interfacial shape distribution of stereom. *κ*_1_ and *κ*_2_ are the maximum and minimum principal curvatures, respectively. **k** Visualization of regions in stereom with curvature distributions shown in (**j**), where purple and green regions correspond to minimal surfaces with zero mean curvature and the saddle surface with the highest distribution density, respectively. **l** An SEM image of the branch surface of stereom.

Sea urchins belong to a group of marine invertebrates, which also include starfish, brittle stars, and sand dollars^12^. Sea urchins are characterized by their dome-shaped body, known as the test, decorated with long, cylindrical spines (Fig. 1a). These often-sharp and spike-like spines act as a defense mechanism against the animal’s predators, including fish, sea otters, starfish, and humans (sea urchins are a delicate cuisine in many cultures)^13^. The spines are in fact highly mobile and can undergo a wide range of movement^14^. This is achieved by the unique articulating joints at the spine-test connection controlled by a ring of active muscle fibers and a collagen-based “catch apparatus”, which can stiffen temporarily to lock and prevent movement of the spines^15^. This allows for independent movement of each spine for controlling the exposure and coverage of the underlying test during protection and locomotion. These biological functions require the sea urchin spines to be stiff, strong, and damage tolerant with the minimum weight possible.

The model system used in this study, *Heterocentrotus mamillatus*, commonly known as slate pencil urchins, has large spines with typical length and diameter of 5–10 cm and 1 cm^16^, respectively (Fig. 1a). Scanning electron microscopy (SEM) of fractured spines reveals their characteristic bicontinuous, highly porous internal microstructure, which is known as stereom^17^ (Fig. 1b). The stereom varies from fully random to highly ordered across different echinoderm species and skeletal parts, representing one of the most remarkable classes of biomineralized structures^17^. The constituent material of stereom is the brittle magnesium-doped calcite (*Ca*_*x*_*Mg*_*1-x*_*CO*_*3*_, *x* = 0.75–0.95) with a small amount of organic materials (1.3 ± 0.3 wt%. See Supplementary Note 1 and Supplementary Fig. 1) in the form of nanoscopic intracrystalline organic inclusions^18, 19^. When viewed in cross-sections, the *H. mamillatus* spines consist of the regular porous stereom and several dark pigmented rings (Fig. 1b, inset and Supplementary Fig. 2). These so-called “growth rings” are resulted from successive thickening during growth, which exhibit low porosity locally in comparison to normal stereom microstructures^20^. The relative density of the stereom structure varies from 10 to 30 vol % in the center region (the blue region in Supplementary Fig. 2a, b) and 20–40 vol % in the edge region (the yellow region in Supplementary Fig. 2a, b)^21, 22^. Their corresponding microstructures are shown in Supplementary Fig. 2c, d. The center region is very localized and only accounts for ~2% of the entire cross-sectional area of sea urchin spines, and the regions between two adjacent growth rings have a regular stereom structure. To avoid the effects of the structural heterogeneity like the very porous center region and the dense growth rings, we focused our investigation on the structure and mechanical properties of the representative stereom within the first growth ring but away from the center.

The stereom structure in *H. mamillatus* resembles a stochastic open-cell foam consisting of interconnected branches and nodes. Figure 1c shows a micro-computed tomography (μ-CT) reconstructed volume of the representative stereom microstructure ($\bar{\rho }$ = 0.37) extracted from an *H. mamillatus* spine. Together with the corresponding rendering of the void space (Fig. 1c, inset), the stereom microstructure exhibits a bicontinuous morphology, where the solid and void phase each form a continuous, interconnected network. We systematically characterized the porous network of both phases by using our recently-developed cellular network analysis algorithm^21, 22^. This algorithm represents the porous structure with a network of skeletonized branches connected by common nodes (Fig. 1d, e), from which both local branch- and node-level information and long-range network organization characteristics can be obtained. The node density of stereom is estimated as 60,000 mm^−3^ (in comparison to 35,000 mm^−3^ for the void phase). The most prominent node types for stereom are three-branched nodes and the average nodal connectivity is 3.3 (Supplementary Fig. 3). This is in stark contrast to the engineering cellular solids fabricated through various foaming processes, where the dominating node type is four-branched due to surface energy minimization^23^. Moreover, the interbranch angles in stereom are 116.9° ± 5.3° and 107.2° ± 7.9° for three- and four-branched nodes, which are close to theoretical values (120° and 109.5°, respectively), suggesting these branches are volume-maximizing^24^ (Supplementary Fig. 4).

The branch length (*L*_*s*_, the physical length of the curved skeleton path between two nodes) in stereom is estimated as 24.8 ± 11.1 μm (Supplementary Fig. 5), which is smaller than that of the void phase (*L*_*v*_, 38.2 ± 13.7 μm) (Supplementary Fig. 6). While the branches in engineering foams have the characteristic three-cusp hypocycloid morphology (also known as Plateau border) resulted from smooth surface joints of adjacent bubbles in the liquid phase^23^, the branch surface in stereom are highly curved. They are best fitted with a second-order polynomial instead of fourth-order for engineering foams^21, 25^ (Supplementary Fig. 7). We further define the local thickness as the diameter of the greatest sphere that can be fully inscribed within the structure^26^ for the solid and void phase (*d*_*s*_ and *d*_*v*_), respectively, as shown in Fig. 1f, g and Supplementary Fig. 8. *d*_*s*_ ranges from 4 to 24 μm, where the high end corresponds to the nodal regions (Fig. 1f). In contrast, *d*_*v*_ ranges from 8 to 40 μm (Fig. 1g). Moreover, as the void space can be regarded as the network composed of nodes and branches, here we further define the thinnest regions of branches in the void space as throats with the diameter, *d*_*t*_ (Fig. 1c). The throats are randomly distributed in the stereom volume without any preferred locations or orientations (Fig. 1h). Moreover, a significant overlap between the distribution of *d*_*s*_ and *d*_*t*_ is observed (57.7%, the area intersected by the probability distributions of *d*_*s*_ and *d*_*t*_, as shown in Fig. 1i and Supplementary Fig. 9). This indicates that the branch thickness is comparable with the throat size, which facilitates jamming of the fractured fragments in the damage band as we will discuss in more detail later. Further quantitative network analysis of both the solid and void phases in stereom is presented in Supplementary Note 2. Another volume with a smaller relative density ($\bar{\rho }$ = 0.27) taken close to the center region was also analyzed, which showed the similar low nodal connectivity (~3.2) and overlap (~51.1%) between the distribution of *d*_*s*_ and *d*_*t*_ (see Supplementary Fig. 10, Supplementary Table 1, and Supplementary Note 3).

The highly curved and smooth surface morphology of stereom has led to previous  postulation that the echinoderm’s stereom microstructure may be minimal surface structure^27^. Indeed, our recent work discovered the first natural triply periodic minimal surface (TPMS) structure in the starfish *Protoreaster nodosus*’ lattice-like stereom^28^. Here we calculated the maximum (*κ*_1_ = 0.11 ± 0.19 μm^−1^) and minimum (*κ*_2_ = −0.08 ± 0.29 μm^−1^) principal curvatures of the stereom surface. As indicated in the interfacial shape distribution plot (Fig. 1j), all surface points of the stereom are characterized by negative Gaussian curvature values (*G* = *κ*_1_•*κ*_2_). Unlike the TPMS structure observed in *P. nodosus*, the mean curvature *H* (= (*κ*_1_ + *κ*_2_)/2) of the sea urchin spine is slightly greater than zero but with a narrow distribution (0.01 ± 0.023 μm^−1^). In addition, the surface with nearly zero mean curvature is located at the nodal regions, whereas the surface with positive values is distributed on the branches, corresponding to the saddle surface (Fig. 1j, k and Supplementary Fig. 11). Moreover, the smoothness of the curved branches is maintained at the nanometer scale, according to the high-resolution SEM reconstruction and atomic force microscopy imaging (Fig. 1l and Supplementary Fig. 12).

**Fig. 2:**
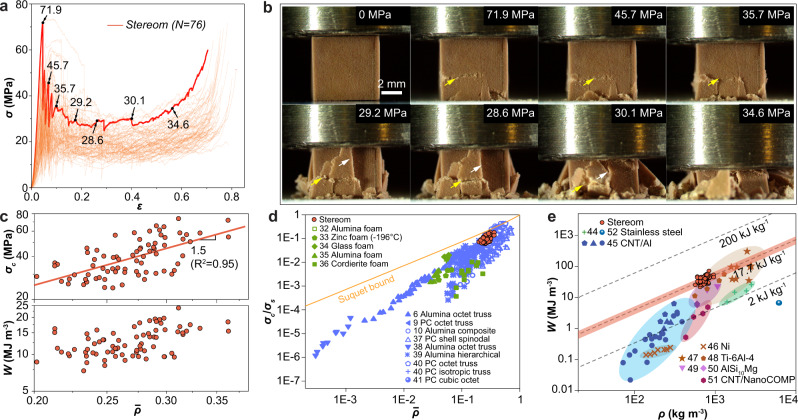
Mechanical properties of the echinoderm stereom from *H. mamillatus* spines. **a** Stress–strain curves of 76 compression tests. **b** Snapshots of deformation stages corresponding to a representative curve in (**a**). The yellow and white arrows indicate the horizontal damage bands and the vertical spallation, respectively. **c** Compressive strength, *σ*_*c*_, and energy absorption capacity, *W*, versus relative density, $\bar{\rho }$. **d** Relative compressive strength, $\frac{{\sigma }_{c}}{{\sigma }_{s}}$, versus $\bar{\rho }$ (*σ*_*s*_: fracture strength of biogenic calcite), in comparison to conventionally^32–36^ and additively^6, 9, 10, 37–41^ manufactured ceramic foams. **e** Energy absorption capacity, *W*, versus density, *ρ*, in comparison to synthetic cellular solids^44–52^. The dashed lines represent different *W*/*ρ* values, and the shaded area highlights the standard deviation of *W*/*ρ* for stereom.

We next conducted uniaxial compression tests on cube-shaped stereom samples (“Ex situ” in Supplementary Fig. 2a) cut from the *H. mamillatus* spines, which reveal the stereom’s remarkable high strength and graceful failure behavior despite its brittle calcite constituent (Fig. 2a, b). The stress–strain curves follow the classic behavior observed in ductile metallic or polymeric foams, which includes a linear elastic regime, a stress plateau regime, and a final densification regime with steeply rising stress^4^ (Fig. 2a). The compressive strength, *σ*_*c*_, is estimated as 40.4 ± 12.4 MPa (*N* = 76), and scales with a relative density as ${\sigma }_{c} \sim {\sigma }_{s}{\bar{\rho }}^{1.5}$ (*R*^2^ = 0.95, Fig. 2c), where *σ*_*s*_ is the strength of biogenic calcite. This result suggests that the stereom structure behaves similarly to a bending-dominated cellular solid^29^. Moreover, with *σ*_*s*_ = 450 MPa for biogenic calcite (Supplementary Note 4)^30, 31^, the stereom exhibits a high relative strength ($\frac{{\sigma }_{c}}{{\sigma }_{s}}$ ~0.1) for $\bar{\rho }$ in the range of 0.2–0.4, which is higher than many conventionally^32–36^ and additively^6, 9, 10, 37–41^ manufactured ceramic foams, and approaches the Suquet bound as the theoretical maximum relative strength for isotropic cellular solids^42^ (Fig. 2d and see further discussions in Supplementary Note 5). Compression tests on samples with the height-to-edge length ratio of ~1.5 following the ASTM C1424 standard^43^ have also been conducted, which exhibits consistent mechanical performance (Supplementary Fig. 13).

Following the initial failure, a relatively constant stress level (ca. 50% of *σ*_*c*_) is maintained during the continuous crushing of stereom samples, leading to the stress plateau regime. Careful examination of the deformation process reveals two main failure modes: the horizontal damage bands and the vertical cracking-induced spallation, as indicated by the yellow and white arrows, respectively, in Fig. 2b. While the spallation leads to stress reduction, the stress level is maintained during the development of horizontal damage bands. To further confirm this, we have conducted compression tests where the stereom sample was constrained in a glass tube in order to minimize the lateral sample expansion resulted from spallation (Supplementary Fig. 14). As expected, the constrained compression tests exhibit less reduction in the plateau stress in comparison to the unconstrained tests. The high stress level in the plateau regime leads to stereom’s remarkable energy absorption capability of 17.7 ± 4.0 kJ kg^−1^, which outperforms many metal- or composite-based foams^44–52^ (Fig. 2e). A slightly lower energy absorption has been observed for spine samples with growth rings, which resulted from a greater reduction in plateau stress due to early spallation from growth rings^53, 54^ (Supplementary Fig. 15).

**Fig. 3:**
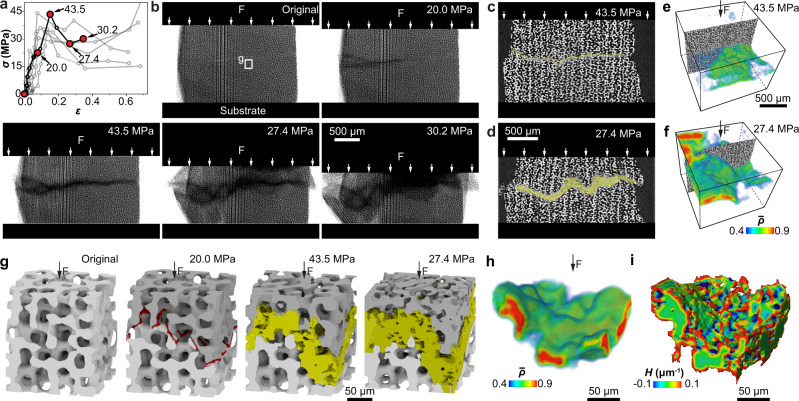
Synchrotron X-ray µ-CT based in situ mechanical analysis. **a** The stress–strain curves of stereom from 11 in situ tests. **b** X-ray projection images of deformation stages corresponding to the curve with highlighted data points in (**a**). **c**, **d** Vertical reconstruction slices and **e**, **f** corresponding 3D renderings of relative density ($\bar{\rho }$), showing the evolution of damage bands in stereom (shaded in yellow in **c**, **d**) at applied stress *σ* = 43.5 MPa and 27.4 MPa, respectively. **c**, **d** Share the same scale bar. **e**, **f** share the same scale bar and color bar. The color in **e**, **f** is set transparent when $\bar{\rho } \, < \, 0.4.$ **g** 3D renderings of a small volume extracted from the test sample in (**b**) showing the deformation process in stereom. The initial cracks and damage band are labeled in red and yellow, respectively. **h**, **i** 3D rendering of the damage band at the applied stress σ = 27.4 MPa in (**g**) in terms of **h** relative density and **i** morphology overlaid with mean surface curvature *H*.

The analysis above indicates that the formation and development of the horizontal damage bands are essential in maintaining high plateau stress for continuous crushing and improved energy absorption. As a comparison, for the synthetic foams and architected foams, spallation occurs instantaneously after the peak stress (see Supplementary Figs. 16, 17 for further details). We next conducted synchrotron-based in situ mechanical measurements to gain a deeper understanding of this intriguing behavior. Similar to ex situ measurements, the small stereom samples (2 × 2 × 1.7 mm^3^, “In situ” in Supplementary Fig. 2a) developed a high stress plateau during compression (Fig. 3a). Corresponding projection images clearly show the formation of damage bands with reduced X-ray intensity, which propagate and enlarge within the sample volume (Fig. 3b). Vertical cross-sectional slices reveal that the damage band is resulted from local microfracture and continuous densification, whereas the rest of the stereom microstructure remains intact (Fig. 3c, d and Supplementary Fig. 18). Quantitative segmentation of the damage bands reveals that the relative density within the damage bands can reach up to 0.9 (Fig. 3e, f, h and Supplementary Fig. 19).

Representative 3D volumes extracted from the consecutive deformation steps further allow us to visualize the structural evolution of the damage band at the individual branch level during crushing (Fig. 3g). A tortuous fracture path was first developed, which dictates the formation and subsequent widening of the damage band due to branch fracture (Fig. 3g, h). The damage band has a rough boundary surface embedded in the surrounding intact stereom, but the roughness is typically within a branch length, as evident by the 3D rendering of the segmented damage band overlaid with the mean surface curvature (Fig. 3i). The locations of damage bands vary among samples, i.e., at sample interior (Fig. 3b) or close to compression platens (Supplementary Fig. 20), which is believed to depend on the local geometrical irregularities of samples.

**Fig. 4:**
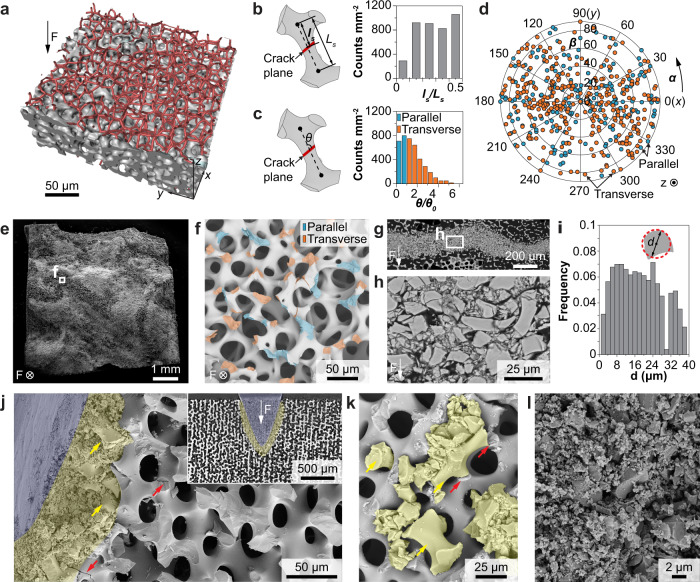
Crack initiation and damage band formation in stereom. **a** 3D reconstruction of the fracture surface (gray) overlapped with the original cellular network (red skeleton). **b** Schematic diagram of crack location (*l*_*s*_*/L*_*s*_) and measured distributions. **c** Schematic diagram of crack orientation (*θ*) and measured distributions. ${\theta }_{0}={{{{{{\rm{atan}}}}}}}({d}_{0}/{L}_{s})$. $\theta /{\theta }_{0}$ ≤ 1: parallel crack; $\theta /{\theta }_{0}$ > 1: transverse crack. **d** The correlation between the crack type and orientation in space. **e** SEM image of a representative compression test sample after crack initiation and **f** a zoom-in view showing the fracture surface with each crack colored in terms of crack type. **g**, **h** Side-view SEM images of damage bands formed during compression tests. **i** Distribution of fractured fragment size shown in (**g**). *d*: equivalent diameters of fractured fragments. **j** SEM image and (inset) corresponding µ-CT reconstruction slice of indentation residues of stereom. **k** Jamming of fractured fragments (shaded in yellow, large fragments highlighted by yellow arrows) by the small throats of stereom immediately after fracture (highlighted by red arrows). **l** SEM image of highly fragmented stereom.

By combining the cellular network analysis with the in situ mechanical data, we are able to uncover how the initial fracture plane develops in the stereom’s porous microstructure. As shown in Fig. 4a, through a customized deep-learning-based damage analysis algorithm (“Methods”), we first identified the fracture plane and then overlaid it with the original skeletonized cellular network. This allows us to determine the locations and orientations of the cracked surfaces for individual branches. The crack location on a branch is defined by the ratio *l*_*s*_*/L*_*s*_, where *l*_*s*_ represents the distance between the crack plane and the nearer node of the fractured branch with length *L*_*s*_ (Fig. 4b). The crack orientation is characterized by *θ*, the angle between the crack plane and the branch, normalized by ${\theta }_{0}={{{{{{\rm{atan}}}}}}}({d}_{0}/{L}_{s})$ (Fig. 4c). *d*_0_ is the minimum diameter on the branch. We further define “parallel” ($\theta /{\theta }_{0}$ ≤ 1) and “transverse” ($\theta /{\theta }_{0}$ > 1) cracks with respect to the branch orientation (Supplementary Fig. 21). Our results indicate that the cracks show no preferred fracture location or orientation (Fig. 4b, c). This is a surprising result as one may expect that fracture should preferably occur at the thinnest location of the branches along the transverse direction. Additionally, the crack planes of individual branches are also randomly oriented with respect to the loading direction, regardless of parallel or transverse cracks (Fig. 4d). This behavior is further supported by direct SEM observations of the fracture surface with randomly-oriented fractured surfaces on individual branches (Fig. 4e, f).

As the in situ X-ray measurements have limited spatial resolution (voxel size, ~0.69 μm), we conducted a further morphological analysis of the damage band via post-mortem electron microscopy characterizations. As shown in the SEM images of a polished surface, the damage band is composed of densely packed fractured stereom fragments (Fig. 4g, h). The boundary between the damage band and intact stereom microstructure can be clearly identified within the distance of one branch length, consistent with μ-CT measurements. The fractured fragments exhibit a large size variation from sub-100 nm up to 40 μm in diameter (Fig. 4i and Supplementary Fig. 22). Notably, the sizes of the large fragments are close to or greater than the throat size of stereom. This indicates that the stereom’s unique bicontinuous structure is able to provide effective local jamming once branches undergo fracture at a critical stress level. This is further supported by performing in situ indentation experiments on stereom samples, which induced the controlled formation of damage bands surrounding the indentation tip (Fig. 4j and Supplementary Fig. 23). More importantly, we can clearly observe that large fragments are jammed by the surrounding structure (yellow arrows, Fig. 4j, k). The small throat opening facilitates the immediate jamming of branch fragments upon fracture, as shown in a carefully preserved fracture surface (Fig. 4k and Supplementary Fig. 24). In the region closer to the indention tip, the fractured fragments have been transformed into densely packed nano-sized fractured particles (blue shaded region in Fig. 4j, l). Continuous loading leads to progressive defragmentation, frictional reorientation, and densification of nanosized grains, contributing to enhanced energy absorption (Supplementary Fig. 25). We also note that beyond the “jamming zone”, random microcracking develops and extends to the surrounding stereom (red arrows, Fig. 4j, k), which contributes to continuous microfracture and jamming upon further loading.

**Fig. 5:**
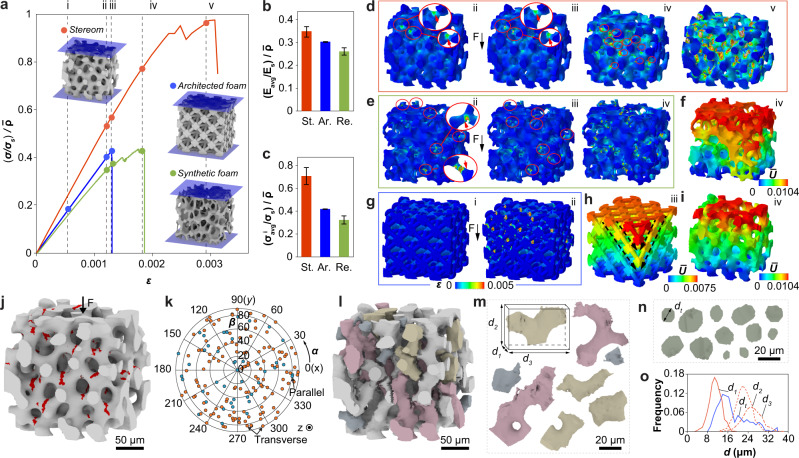
Computational modeling of the mechanical performance of stereom. **a** Simulated compressive stress–strain curves for stereom ($\bar{\rho }$ = 0.37), reticulated foam ($\bar{\rho }$ = 0.29), and octet truss ($\bar{\rho }$ = 0.34). The stresses are normalized by *σ*_*s*_ and $\bar{\rho }$. Comparison of **b** stiffness, **c** strength, and **d**–**i** fracture behavior of these three structures. Error bars in **b**, **c** indicate mean ± standard deviation. **d**, **e**, **g** Show the distribution of maximum principal strains at applied strains of (i) 0.0007, (ii) 0.0012, (iii) 0.0013, (iv) 0.0018, and (v) 0.0029. The circles and arrows mark the locations of microcracks. **f**, **h**, **i** Normalized displacement ($\bar{U}$) fields at stages corresponding to (**a**), where the displacement *U* is normalized by the characteristic length *S*_*v*_ (the sum of average branch diameter and average throat diameter) of each model. The dashed arrows mark the fracture of the octet truss along the weak plane. **j** Simulated distribution of microcracks (highlighted in red) in stereom, and **k** corresponding distribution of crack orientation. **l** Registration of individual fractured fragments based on simulation results. **m** Representative isolated fragments with each fragment measured by three orthogonal dimensions (${d}_{1} < {d}_{2} < {d}_{3}$). **n** Representative throats with the size defined by the diameter *d*_*t*_. **o** A comparison between the fragment sizes (*d*_1_, *d*_2_, *d*_3_) and the throat size (*d*_*t*_) based on the simulated volume (**l**).

The mechanical experiments above demonstrate that the formation of densely packed damage bands via microfracture and jamming is essential in enhancing the energy absorption capability of stereom, which has not been observed in engineering ceramic cellular solids (Supplementary Figs. 16, 17). To understand the mechanism, we next conducted a systematic finite element (FE)-based fracture modeling to compare the mechanical response between the natural and the artificial systems (Fig. 5). Here the engineering foams include a stochastic open-cell alumina-based foam ($\bar{\rho }$ = 0.29) and silica-based octet truss lattices ($\bar{\rho }$ = 0.26, 0.34) fabricated from the conventional replication process and additive manufacturing, respectively (Supplementary Figs. 26, 27). The 3D models of the reticulated and architected foams were obtained via laboratory μ-CT measurements (Supplementary Figs. 28, 29 and “Methods”). Although the modeling cannot capture the full plateau regime due to the convergence issue resulting from significant cracking, the stereom exhibits superior relative stiffness ($\frac{E}{{E}_{s}\bar{\rho }}$), relative strength ($\frac{{\sigma }_{c}}{{\sigma }_{s}\bar{\rho }}$), and total energy absorption (*W*) simultaneously (Fig. 5a–c, Supplementary Fig. 30, and Supplementary Tables 2, 3). The computational modeling also allows us to investigate the differences in stress distribution and damage evolution in these material systems. As ceramics fail more easily under tensile stresses, here we compare the distributions of maximum principal stress, which reveal that the stereom exhibits a much lower stress concentration in comparison to engineering foams (Supplementary Fig. 31). This is because the reticulated and architected foams often have very thin branches and/or sharp curvatures (Supplementary Figs. 32, 33 and also see Supplementary Note 6 for further discussions). The stress concentrations in these engineering foams are expected to be even higher in experiments due to the presence of microscopic defects, such as sintering holes in reticulated foams after template removal, microscopic porosities, and surface roughness^11, 55^ (Supplementary Fig. 34). The high stress concentrations lead to the reduced strength observed in engineering foams (Supplementary Figs. 16, 17).

Moreover, the damage process in stereom is a gradual process: microcracks initiate locally and then gradually populate to neighboring branches (Fig. 5d and Supplementary Fig. 35). This leads to a progressively enlarged damage zone, consistent with previous in situ observations (Fig. 3a, b). Remarkably, during this microcracking process, the stereom remains structurally intact and sustains increasing loading (Fig. 5a, d, i). In contrast, the reticulated foam develops multiple cracks throughout the entire volume simultaneously due to the presence of high stress concentrations (Fig. 5e). Further loading quickly results in crack coalescence and structural collapse (Fig. 5f and Supplementary Fig. 36). For the architected ceramic lattice, the stretching-dominant behavior and stress concentrations at the nodal regions lead to simultaneous crack initiation along specific crystallographic planes, which causes brittle, cleavage-like fracture at critical loads^6, 8^ (Fig. 5g, h and Supplementary Fig. 37).

With the simulated fracture stereom microstructure, we further confirm that the cracks show no preference in locations and orientations at individual branch level, consistent with experimental observations (Fig. 5j, k and Supplementary Fig. 38). Moreover, by extending the crack planes to fracture through individual branches, we are able to extract and register individual fractured fragments computationally (Fig. 5l, m). Consistent with our experimental results, the fractured fragments display larger sizes in comparison to the throat openings in stereom (Fig. 5n, o and Supplementary Fig. 39). This further supports the jamming-induced damage localization and energy dissipation observed experimentally.

**Fig. 6:**
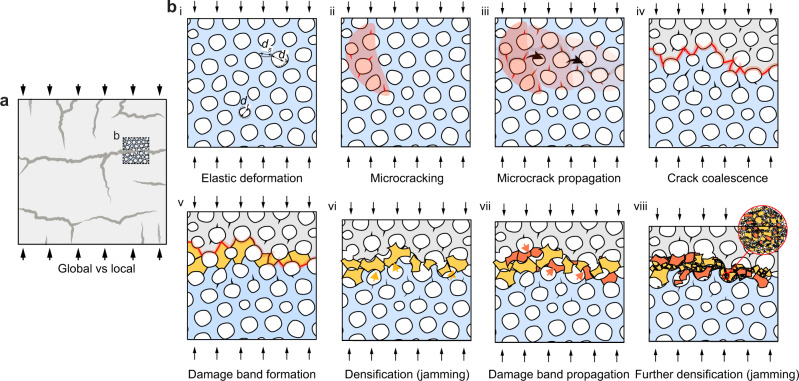
Correlation between the macroscopic response and the microstructure of stereom. **a** The deformed sample with a small volume cropped to show the failure process in (**b**) from stage i to stage viii. **b** Microstructure-level deformation exhibits elastic deformation (i), microcracking (ii), micro-crack propagation (iii), crack coalesce (iv), damage band formation (v), densification (jamming) (vi), damage band propagation (vii), further densification (jamming) (viii).

Combining the experimental and computational results, a summary of the fracture process and the origin of high damage tolerance and energy dissipation of the sea urchins’ stereom is depicted in Fig. 6. On the macroscopic level, the formation of localized and densely packed damage bands leads to gradual crushing and efficient energy dissipation, whereas the vertical cracking-induced spallation results in stress reductions in the plateau regime (Fig. 6a). By minimizing spallation with lateral constraints during compression, the energy absorption of stereom can be enhanced (Supplementary Fig. 14).

At the microscopic level, the stereom’s unique bicontinuous cellular network with smooth surface curvatures offers several significant advantages in comparison to the engineering ceramic foams upon loading. During elastic deformation (Fig. 6b, stage i), the stress concentration on the stereom is reduced due to the absence of very thin branches, sharp curvature transitions, microscopic porosities, and surface roughness, which are hard to eliminate in engineering ceramic foams with current fabrication technologies^11^ (Supplementary Note 6). Moreover, the defect size of biogenic calcite is much smaller than the branch size of stereom and thus entails a high strength (see further discussions in Supplementary Note 7), which works synergistically with the bicontinuous geometry of stereom to achieve high mechanical efficiency. Beyond the elastic limit, both the experimental results (Fig. 4a–d) and FE analysis (Fig. 5j, k) show that dispersed microcracks are initiated randomly in the stereom due to low-stress concentration on the stereom surface (Fig. 6b, stage ii). These microcracks propagate (Fig. 6b, stage iii) and coalesce (Fig. 6b, stage iv) as their density and sizes increase during loading, which further leads to the fracture of branches and formation of fractured fragments (Fig. 6b, stage v). The combined evidence from the in situ observation of the deformed volume (Fig. 3g), SEM examination of the residues (Fig. 4j), and FE-based fracture simulations indicate that these fractured fragments can be easily jammed by the small throat openings in the stereom (Fig. 6b, stage vi). This is facilitated by the fact that the branch thickness is comparable with or larger than the throat sizes in the void space. This jamming process leads to the formation of a densely packed horizontal damage band, where the load can be redistributed to the entire fracture surface instead of concentrating on local nodes and struts. As a result, the stress concentration in adjacent stereom microstructure is reduced, resulting in distributed microcracks propagating to the surrounding intact stereom microstructure (Fig. 6b, stage vi). This allows for further widening of the damage band through local jamming without catastrophic crushing (Fig. 6b, stage vii). Therefore, the strength of the deformed stereom is not significantly reduced during the formation and propagation of damage bands. Further loading leads to continuous defragmentation of the fractured branches in the damage band, where the formed micro-/nano-sized grains undergo reorientation, friction, and deformation, leading to further energy dissipation (Fig. 6b, stage viii).

## Discussion

Our results suggest that the formation of damage bands enabled by microfracture and local jamming is essential in enhancing the energy absorption capability of the echinoderm’s stereom as a ceramic cellular solid. Several structural design strategies are worth highlighting here. Firstly, both cellular network characteristics (low nodal connectivity) and mechanical behavior (${\sigma }_{c} \sim {\sigma }_{s}{\bar{\rho }}^{1.5}$) indicate that the stereom is a bending-dominated structure^29^. The stretching-dominated lattices, despite being stiffer and stronger compared to bending-dominated cellular solids of the same relative density, often fail under “hard” modes (tension, compression) rather than “soft” ones (bending)^56^. This results in brittle opening or collapse of the branches, leading to more severe post-yield softening^6, 8^. In addition, the “hard” mode failure makes them less advantageous for energy absorption in comparison to stereom, which exhibits a stress–strain curve with a long, flat plateau. Secondly, the bicontinuous morphology of stereom also carefully controls the morphology of the void space by eliminating large, grain-like void cells, which is common in engineering foams fabricated through various foaming processes^11, 55^ (see an example in Supplementary Fig. 40). The large void cells in engineering foams are less efficient in providing jamming of fractured branches. Thirdly, we note that stereom often avoids the highly regular lattice structures, which eliminates cleavage-like brittle fracture along specific crystallographic planes at the lattice level^6, 8^. A recent work revealed that the ‘tailored disorder’ as observed in many natural cellular materials leads to enhanced fracture resistance in comparison to fully ordered cellular structures^57^. Moreover, our recent work identified a unique diamond-TPMS lattice structure in the starfish *P. nodosus*, in which the organism utilizes lattice defects, such as dislocations, to suppress such catastrophic failure^28^. Fourthly, the stereom in sea urchin spines exhibits remarkable control of surface roughness and defects, which reduces stress concentration at the structural level significantly. Lastly, unlike engineering ceramic foams that are based on sintered microscopic grains in the branches and nodes, the stereom is constructed based on a continuous single-crystalline biogenic calcite^19, 58^. Similar to other calcitic biominerals, the stereom exhibits the so-called conchoidal fracture behavior as observed in glassy materials rather than brittle cleavage along the {104} planes as in geological calcite^19, 53, 58^. This behavior, which has been shown resulted from the strengthening and toughening effects from the nanoscale intracrystalline organic inclusions in biogenic calcite^19, 31, 59^, facilitates more extensive microcracking and defragmentation during the damage band formation and densification, which improve the energy absorption of stereom further^31, 60^.

## Methods

### Samples

The dried spine specimens from the sea urchin *H. mamillatus* were purchased from Etsy, and no special treatment was applied. The synthetic reticulated ceramic foam was purchased from SELEE®CS-X (25 PPI, Selee, United States). The silica-based architected lattice was produced through a stereolithography-based 3D printing process using a commercial desktop 3D printer (FormLabs 2; Formlabs Inc., MA, USA). The ceramic resin developed by FormLabs was used, which contains ceramic (primarily silica) particles mixed with a photocurable polymer matrix. The ceramic/polymer composite was first printed and then underwent a sintering process following the recommended sintering process (maximum temperature: 1271 °C) provided by the manufacturer to obtain the final ceramic parts.

### Electron microscopy

SEM imaging was performed on dried specimens, including fractured surfaces, compression residues, and indentation residues. Before imaging, all specimens were coated with Pt/Pd to reduce charging effects. The fractured surfaces and compression residues were imaged using a Quanta 600 FEG Environmental SEM (FEI, OR). The indentation residues were imaged using an LEO 1550 Field Emission SEM (Zeiss, Munich). The SEM images were taken at acceleration voltages of 2–10 kV and working distances of 4–10 mm.

### AFM measurement and analysis

The noncontact topography measurements were performed on the branch surface of the stereom with a commercial AFM (XE7, Park System, Santa Clara, CA) using a non-contact AFM mode. The AFM tip (NANOSENSORS, PPP-NCHR) was used. The AFM topography was measured using the higher image resolution (512 × 512 pixels) with a field view of 9 × 9 μm^2^. The scan rate along *X* axis per line was 0.1 Hz. The image processing was performed through commercial software (XEI, Park systems).

### Ex situ mechanical tests

Cube-shaped stereom samples from *H. mamillatus* spines (edge length: 5–8 mm, *N* = 76) and alumina-based reticulated foams (edge length: 10 mm, *N* = 5) were cut using a low-speed diamond wheel saw (MTI Corporation, CA, USA). Uniaxial, quasi-static compression experiments were performed on these samples at a displacement rate of 0.2 mm min^−1^ with a universal testing machine (Instron 5984; Instron, MA, USA). Constrained compression tests were conducted by placing stereom samples inside a glass tube (thickness, 1.75 mm; Friedrich & Dimmock Inc., NJ, USA). The videos of tests were recorded at a frame rate of 500 frames min^−1^ for additional analysis of the deformation processes.

### Synchrotron-based in situ mechanical tests

The in situ mechanical tests based on synchrotron µ-CT were conducted at the beamline 2BM from Advanced Photon Source, Argonne National Laboratory. A customized in-situ loading device was used for compression and indentation tests of stereom samples, through which the samples were mechanically loaded while allowing for X-ray imaging through an X-ray transparent window. The test samples typically measured 2.2 × 2.2 × 1.7 mm^3^. A monochromatic beam with an energy of 27.4 keV was used for the measurements. For compression tests, the samples were compressed by steel plates through a stepwise fashion (typical step size, *ca*. 0.1 mm). For indentation tests, an alumina conical tip was used to induce localized deformation (half apex angle, 19°; tip diameter, 120 μm). The beamline was equipped with a single-crystal LuAg:Ce scintillator for converting X-ray into visible light, which was further magnified by using 2× or 5× long-working distance objective lenses. For a typical scan, 1500 projection images were acquired during a 180° rotation with an exposure time of 0.1 s projection^−1^ (*ca*. 2.5 min for a single tomography scan). The projection images were collected using a PCO-Edge high-speed CMOS detector (2448 × 1024 pixels), which resulted in a typical voxel size of 1.725 or 0.69 µm depending on the objective lens used. The reconstruction of the obtained µ-CT data was conducted with the open-source software Tomopy^61^. The reconstructed data was used for 3D volume rendering and quantitative analysis via a combination of methods, such as Avizo (Thermo Fisher Scientific, MA, USA), Fiji^62^, open-source software Blender (www.blender.org), and customized MATLAB codes.

### Quantitative cellular network analysis

The porous cellular network of stereom and the corresponding inverse structures were analyzed by using a quantitative network analysis algorithm developed by our group recently^21, 22^. Briefly, the analysis pipeline includes tomography data reconstruction, segmentation, network registration and representation, and multiscale network quantification. Representative volumes of stereom were acquired from the synchrotron µ-CT data and corresponding void structures were obtained by inversing the original data. In this algorithm, the bicontinuous porous structure was skeletonized and represented as a network composed of branches connected with common nodes. Subsequently, the node- and branch-level information, such as node type, node orientation, branch length, branch thickness, branch orientation, and branch profile, can be quantified. To avoid edge effects (e.g., incomplete branches), nodes and branches within 26 μm from the boundaries were excluded from the analysis.

### Deep learning-based damage analysis of in situ data

We developed deep learning (DL)-based algorithms to automatically identify, register, and analyze the deformation-induced damages in the tomography data obtained from synchrotron in-situ measurement. We particularly focused on two types of damages, i.e., microcracks and damage bands at the initial and later stages of deformation, respectively. For the analysis of initial microcracks in this work, by taking advantage of these high-contrast features at the individual branch level, we utilized a DL-based local feature recognition algorithm for high-fidelity detection. First, a small set of reconstruction slices (ca. 15 slices) were carefully examined by expert image readers to register individual microcracks in small regions. These manual labels were used as the ground truth to train a deep convolutional neural network (DNN) for automatic detections. Here we adopted a simplified VGG16 network to process the input of three-channel stacked local tomography slices composed of 32 × 32 × 3 voxels centered around each crack candidate location. Each candidate was sliced and examined in three orthogonal orientations and confirmed as a crack by a majority vote. After the individual crack surface was registered, an overlapping comparison was implemented between the cracked region and the original undamaged volume through a digital volume correlation (DVC) analysis. This allows us to (1) reconstruct the 3D crack morphology, (2) identify the branches in the original structure that were finally cracked, and (3) quantitatively register the crack location and orientation as referenced by the hosting branch. We registered the crack location in the undamaged structure by maximizing the correlation coefficient between the two volumes. The remaining different voxels between the overlapped volume on the branch were then registered as the crack voxels. Connected cracks that extended across multiple branches were segmented and each crack was assigned to the nearest branch. The center of each segmented crack plane was identified by averaging all voxel locations in the crack, and the crack plane orientation was determined by the minimum principal component with principal component analysis. Further details regarding crack detection and analysis can be found in a previous work^63^. For the analysis of damage bands at the later stage of deformations, we adopted a U-net architecture in a DNN to learn the features of the fragments from the manual labeling. The trained DNN then segmented the damage bands in semi-3D manner^64^, by processing input of large-scale tomography slice stacks (2048 × 2048 × 5 voxels) in all three orientations. Next, a majority vote of segmentation results from the three orientations yielded a high-fidelity fragmentation identification in 3D. An overlapping correlation analysis was implemented for the fragmented region with the undamaged cellular structure following a similar strategy used for crack analysis. This treatment allows the identification of the boundary of damage bands and the registration of individual branches supporting the damage bands. In addition, the individual fragments within the damage bands were segmented and quantified in 3D by their volume and aspect ratio, from which the relative density of the damage band was quantified.

### Laboratory X-ray tomography and data analysis

The reticulated foams with 25 PPI size and the 3D-printed architected lattices were scanned using a high-resolution laboratory µ-CT Skyscan system (Model 1172; Skyscan, Aartselaar, Belgium). The X-ray tube was operated at 80 kV and 100 µA. Each tomography scan consisted of 273 projection images with an exposure time of 5.082 s per projection and was acquired over a 180° sample rotation. Projections were collected using a Hamamatsu camera (1280 × 1024 pixels) and the resulted isotropic voxel size was 17.42 µm. The reconstructed data was used for 3D volume rendering and quantitative analysis via a combination of methods, such as Avizo (Thermo Fisher Scientific, MA, USA), Fiji^62^, open-source software Blender (www.blender.org), and customized MATLAB codes.

### Nanoindentation

The dried *H. mamillatus* spines were cut into cylindrical sections (*ca*. 10 mm), embedded in Epofix™ epoxy (Electron Microscopy Sciences, PA, USA) and subsequently cured at room temperature. The samples were then polished on a polishing machine (MultiPrep^TM^ System; Allied High Tech Products Inc., CA, USA) with diamond lapping films stepwise (15, 9, 6, 3, and 1 µm), and finally with 40 nm colloidal silica suspension on a polishing cloth. Load-controlled nanoindentations were performed on the polished sample surface and the damage bands in the indentation residues using a Berkovich diamond tip (trigonal pyramid, semi-angle = 65.3°) on a Micro Material indentation system (Nano Test Vantage Platform 4, Wrexham, UK). The maximum loads varied from 5 to 25 mN. Typical load functions included successive loading (15 s), holding (10 s), and unloading (15 s) sections. The thermal drifting was monitored when the load was unloaded to 10% of the maximum force for 30 s. The hardness *H*_*O-P*_ and indentation modulus *E*_*O-P*_ of the biogenic calcite and the damage band were quantified based on the standard Oliver-Pharr (O-P) methodology^65^.

### Thermogravimetric analysis

The sea urchin spines were first cut transversally into cylindrical sections, which were further ground into powders manually using a grinding mortar and pestle. The powders obtained were then placed in the platinum pan for thermogravimetric analysis (Q50 Thermogravimetric Analyzers, TA Instruments). The experiment was carried out under N_2_ with a flow rate of 40 mL min^−1^ from RT to 900 °C. The controlled heating profile starts with a ramp rate of 5 °C min^−1^ from room temperature to 110 °C and hold for 15 min before another ramp at a rate of 10 °C min^−1^ until 900 °C.

### Finite element analysis

Abaqus/Standard 2016 (Dassault Systems, Vélizy-Villacoublay, France) was implemented to simulate the mechanical response of five structures with the same non-dimensional length (5-characteristic length *S*_*v*_ in each dimension. *S*_*v*_ is defined as the sum of average branch diameter and average throat diameter) from the stereom ($\bar{\rho }$= 0.37, 0.27), the reticulated foam ($\bar{\rho }$ = 0.29) and the architected foam ($\bar{\rho }$ = 0.26, 0.34) under uniaxial compression. The geometry of all the models was based on µ-CT reconstruction. The constructed models were then discretized using tetrahedral elements and the constituent material was set with a modulus of 109 GPa and a Poisson’s ratio of 0.291^66^. For each model, two independent simulations were performed, i.e., the general statics-based simulation and the explicit dynamic module-based simulation, which were used to study the elastic response and fracture behavior of the corresponding cellular structure respectively. More specifically, the static simulations were force controlled, and the stress/strain distributions of different structures were exported at the same applied compressive stress of 6.02 MPa for a fair comparison. In contrast, the explicit simulations performed to capture the micro-cracking processes were based on explicit simulation with general contact and element deletion. In these explicit fracture simulations, the loading displacement rate is controlled ensuring that the inertial effect (and the kinetic energy of the structure before fracture happens) is negligible, thus corresponding to a quasi-static loading condition. A brittle cracking model and element deletion were implemented to capture the fracture process. In the simulation, the strain rate of loading was controlled to guarantee a quasi-static condition. Cracks were initiated when the maximum principal tensile stress exceeded 100 MPa^30, 31^. The fracture energy of forming a unit area of the crack surface in Mode I, *G*_If_, was used as the criteria of element deletion to avoid unreasonable mesh sensitivity^67^. A linear shear retention model was also included, considering the reduction of post-cracked shear modulus as the crack opened. In the explicit simulations, the applied pressure of loading was set as *P* = 6.02 MPa.

## Supplementary information

Supplementary Information

## Data Availability

The data support the key findings generated in this study are provided in the Supplementary Information. All raw data generated during the current study are available from the corresponding author L.L. upon request.
