# Supplementary material for: High strength and damage-tolerance in echinoderm stereom as a natural bicontinuous ceramic cellular solid
Source: Nat Commun. 2022 Oct 14;13:6083. doi: 10.1038/s41467-022-33712-z (PMC9568512; doi:10.1038/s41467-022-33712-z)
Supplement: Supplementary file 1 — Supplementary Information [file 41467_2022_33712_MOESM1_ESM.pdf]

Supplementary Information for

High strength and damage-tolerance in echinoderm stereom as a natural bicontinuous ceramic cellular solid

Ting Yang[1], Zian Jia[1], Ziling Wu[2], Hongshun Chen[1], Zhifei Deng[1], Liuni Chen[1], Yunhui Zhu[2]

and Ling Li[1,*]

[1]Department of Mechanical Engineering, Virginia Tech, Blacksburg, VA 24061, USA.
[2]Department of Electrical and Computer Engineering, Virginia Tech, Blacksburg, VA 24061, USA.

* Corresponding author. Email: lingl@vt.edu

This PDF file includes:

Supplementary note 1-7

Supplementary Fig. 1 - 41

Supplementary Table 1 – 5

Supplementary References (1-62)

Supplementary note 1

Quantification of organic contents through thermogravimetric analysis (TGA) measurements

To quantify the total amount of water and organics in the sea urchin samples, TGA experiments were performed up to the temperature of 900 °C (Supplementary **Fig. 1**). At this temperature, the mineral phase of the sea urchin spine samples ($Mg_xCa_{1-x}CO_3$) is expected to be decomposed into MgO and CaO completely[1]. The decreasing trend of the sample weight in the TGA curves is correlated with the consecutive decomposition process of the sea urchin spine samples. Based on published results of the TGA experiments on the sea urchin samples (spines and test plates of *Paracentrotus lividus*)[1], the profile of weight loss can be divided into three stages: water loss (room temperature to 110 °C), deconstruction of organic macromolecules (accompanied with CO_2 emissions) between 110 – 450 °C, and decomposition of the mineral phases at temperatures between 450 – 800 °C. It is noted that other processes might also be involved in the heating process, such as ACC to calcite transition (at ca. 200 °C with no significant change in weight), while the rate of weight loss might also vary during each temperature range[1]. Also note that the above-identified temperature ranges of different decomposition processes may overlap. For instance, the biogenic calcite with rich Mg-substitution decomposes at a lower temperature (at ca. 400 °C)[2,3]. Here, we approximate the TGA profiles of weight loss following the published results on sea urchin spines[1], and consider that in the temperature range from 110 °C to 450 °C, the measured weight reduction is due to the weight loss of organic content[1]. Based on nine measurements (Supplementary **Fig. 1**), the weight percentage of the organic macromolecules in sea urchin spine samples is estimated to be 1.3 ± 0.3 wt%.

Supplementary note 2

Cellular network analysis of the original stereom and the inverse structures

The representative volumes of stereom and the corresponding void (inverse) structures (244 μm × 244 μm × 244 μm) were skeletonized and represented as networks composed of branches connected with common nodes (see Methods). To avoid edge effects (e.g., incomplete branches), nodes and branches within 26 μm from the boundaries were excluded from the analysis. A series of structural descriptors at the node and branch level were defined[4]. At the node level, the nodal connectivity in stereom and the inverse structure is determined by the number of branches connected to the node. For nodes with three and four connecting branches, the interbranch angle is defined in the same way as in reference[5]. For instance, for nodes with three connecting branches, three interbranch angles and the average interbranch angle are represented as $\gamma_{3,k}$ (k = 1, 2, and 3) in ascending order and γ_3, respectively. Similarly, for nodes with four connecting branches, the minimum, maximum and average interbranch angle are represented by $\gamma_{4,min}$, $\gamma_{4,max}$, and γ_4, respectively. At the branch level, the branch length L_s, L_v and branch distance L_d, L_{vd} are the length of the curved path and Euclidean distance between two nodes for the stereom and the void structure, respectively. Length ratio L_s/L_d and L_v/L_{vd}represents tortuosity. Branch morphology (radius) r is the profile of branch surface which is quantitatively described by a second-order polynomial. For individual branches in stereom and the inverse structures, $r_s(l)$ and $r_v(l)$ are the radius of an equivalent circle with the same area of the intersecting profile (which is between the cross-sectional plane perpendicular to the branch at the sampling point and branch surface at a given location of l) for the stereom and the void structure, respectively. The radius $r_s(l)$ and $r_v(l)$ are normalized by the thinnest radius r_{so} and r_{vo}, respectively. The location l is normalized by branch length L_s and L_v, respectively.

For the stereom volume, nodes with three connecting branches are the dominating node type. The counts ratio of nodes with 3, 4, 5, 6 connected branches is 271:131:8:2 (Supplementary **Fig. 3a-d**). The average nodal connectivity for the stereom structure is 3.3. While in the inverse structure, the average nodal connectivity is 4.6, where the count ratio of nodes with 3, 4, 5, 6, 7, 8, 9, 10 connected branches is 64:65:57:42:10:4:1:2 (Supplementary **Fig. 3e-h**). The average interbranch angle for three-branched nodes (γ_3) and four-branched nodes (γ_4) in stereom are 116.9° $\pm$ 5.3° and 107.2° $\pm$ 7.9°, respectively (Supplementary **Fig. 4a,b**), which are close to the ideal angle 120° and 109.5°, respectively, similar to trabecular bone[7]. The average interbranch angles for three-branched nodes (γ_3) and four-branched nodes (γ_4) in the inverse structure are 108.4° $\pm$ 12.3° and 101.8° $\pm$ 11.1°, respectively (Supplementary **Fig. 4c,d**).

The branch length L_s, branch distance L_d, and length ratio L_s/L_d in stereom (L_v, L_{vd}, and L_v/L_{vd} in the inverse structures) are 24.8 ± 11.1 μm, 20.5 $\pm$ 8.0 μm, and 1.19 $\pm$ 0.12, respectively (38.2 ± 13.7 μm, 30.5 $\pm$ 9.7 μm, and 1.26 $\pm$ 0.23, respectively) (Supplementary **Fig. 5**,**6**). For individual branches in stereom and the inverse structure, the fitted normalized branch profiles are $\bar{r}_s = 1.82\bar{l}^2 - 0.006\bar{l} + 1$ ($R^2 = 0.93$) and $\bar{r}_v = 0.75\bar{l}^2 - 0.084\bar{l} + 1$ ($R^2 = 0.98$), respectively (Supplementary **Fig. 7**).

Supplementary note 3

Structure analysis of the stereom with relative density ~27%

We conducted an additional network analysis of volumes taken within or close to the center region in comparison to the more representative volumes taken from the edge region used in the main text. Here, for example, we analyzed a volume with a relative density of 27% (in comparison to 37% used in the main text). In this volume, the node density is 40,000 mm^{-3} for the stereom and 40,000 mm^{-3} for the void phase. The dominant node type is the three-branched node with an average nodal connectivity of ~3.2 (Supplementary **Fig. 10c,d**). An overlap of 51.2% between the local structure thickness and the throat size also has been observed (Supplementary **Fig. 10e-h**), indicating the structure thickness is comparable to the throat size. These structural characteristics are comparable with those from the representative volume in the main text (Supplementary **Table 1**). Although we cannot directly conduct mechanical tests on these more porous regions due to their very small volume, our finite element analysis revealed a similar uniform stress distribution on the stereom (Supplementary **Fig. 31-33**). In addition, microcracks initiate locally and then gradually populate to neighboring branches, consistent with the observations in the representative volumes (Supplementary **Fig. 35b**).

Supplementary note 4

Intrinsic strength of biogenic calcite

Due to the small sizes and complex geometries of biogenic minerals, it is challenging to directly probe the strength of biogenic calcite, particularly for stereom structures, through experimental methods. Nanoindentation represents an important approach to quantify the mechanical properties of materials at the nanometer scale. Previous studies indicate that, for materials with $\sigma_Y/E < 0.02$,

$$H \approx 2.8\sigma_Y \quad (1)$$

where σ_Y is the yielding strength, H and E are the hardness and modulus[6], respectively. Thus, the compressive yielding strength could be estimated through this relationship and the hardness values could be measured from nanoindentation. In addition, other methods are evolved in determining the intrinsic properties of biogenic minerals, such as micro-pillar compression on Focused-Ion Beam (FIB) milled pillars[7,8], which could approximate the uniaxial compression loading and give strength measurements of yielding strength. For brittle solids, the crushing strength can be more easily measured and is roughly 10 times the tensile strength. Therefore, the failure strength of biogenic calcite can be estimated as 1/10 of the measured/estimated compressive strength. In addition, our recent study also developed a micro-bending method to directly quantify the intrinsic bending performance of prismatic biogenic minerals in mollusc shells, which quantified the bending modulus and failure strength/strain[9]. Supplementary **Table 4** summarizes the yielding and failure strength for different biogenic calcite systems, either acquired through direct experimental measurements or estimated from measured hardness by using Equation-1. Based on this comprehensive literature review and our previous results, the failure strength of biogenic calcite is estimated to range from 63 MPa (from the indentation tests on *Paracentrotus lividus* spine[8]) to 450 MPa (from micro-bending tests on *Atrina* prism[9]).

Supplementary note 5

Comparison of relative strength between the stereom and engineering ceramic foams

The relative strength σ_s of the stereom can be obtained by normalizing the stereom's compressive strength with the failure strength of biogenic calcite in Supplementary Note 4. Here, we used the failure strength[9] of 100 MPa and 450 MPa, which were estimated by indentation tests on *H. mamillatus* spine and measured by micro-bending tests on *Atrina* prism[9].

The Suquet upper bound (relative strength σ_{SU}/σ_s) represents the theoretical strength limit of isotropic cellular solids. The Suquet bound is expressible as a function of the relative density $\bar{\rho}$ in Equation-2[10]:

$$\frac{\sigma_{SU}}{\sigma_s} = \frac{2\bar{\rho}}{\sqrt{4 + \frac{11}{3}(1 - \bar{\rho})}} \tag{2}$$

The Suquet bound of the cellular solids with relative density 0.2 and 0.4 is 0.15 and 0.29, respectively. The relative strengths of stereom are 0.42 ± 0.12 and 0.09 ± 0.03 when the failure strength of 100 MPa and 450 MPa are used, respectively, corresponding to $191 \pm 45\%$ and $43 \pm 10\%$ of the Suquet bound (Supplementary **Fig. 41a**). Therefore, the stereom, as open-cell bending-dominated structures, exhibits a high relative strength that outperforms many stretching-dominated ceramic lattice structures (Supplementary **Fig. 41b**).

Mechanical size effects have been recently frequently utilized to enable nanolattices such as alumina[11] and glassy carbon[12] octet lattices, pyrolytic carbon isotruss[13], and alumina ceramic composites[14] to achieve high strength by significantly improving the corresponding failure strength of the constituent bulk solids. However, the relative strengths of these materials are well below the Suquet bound[10] (Supplementary **Fig. 41b**). The deformation mechanisms, including both stretching-dominated and bending-dominated behaviors, are the main factors that affect the mechanical efficiency, with the stretching-dominated structures being stiffer and stronger[15]. The strength of the ideal analytical models of stretching-dominated structures (such as octet truss and isotropic truss lattices) are limited to half the Suquet upper bounds, with the octet truss also being anisotropic[16–18]. In practice, the stress concentrations at nodes and nodal bending further reduce their actual performance[19], to around 25% and 20% of the Suquet upper bound. More importantly, the manufacturing imperfections may further reduce the structure strength[20]. As discussed in Supplementary Note 6, the bicontinuous stereom structure with smooth surface curvatures reduces stress concentrations efficiently and contributes to the observed high strength. Currently, few open-cell structures are capable of reaching the Suquet upper bound in strength[21,22]. The strength of some closed-cell foams, which consist of plates or membranes arranged in the closest packed planes of crystal structures, are reported to reach the Suquet upper bounds[23]. For example, the cubic+octet (CO) plate lattice reaches the Suquet upper bound when approaching 0% relative density and remains within 90% of the bound at higher relative density[23].

Supplementary note 6

Additional discussion on the relationship between strength and surface curvature

In this supplementary note, we further discuss how surface curvature affects the strength of the stereom. The statistical distribution of surface curvature shown in Supplementary **Fig. 32** includes two scales of geometric information, i.e., the feature size of the branch (diameter) and the feature size of surface roughness/defects of the structure. To distinguish these two feature scales, the Gaussian curvature is normalized by the characteristic length of the corresponding structure, S_v, which is the sum of average branch diameter and average throat diameter. The statistical distributions of S_v are shown in the *x*-axes of Supplementary **Fig. 32**. Because the branch size is roughly 1/5 of the unit cell, a normalized Gaussian curvature around 25 represents the surfaces depicted by the branch thickness of the structure. In contrast, Gaussian curvature whose absolute value is way greater than 25 (e.g., 400) represents regions of feature size much smaller than the branch thickness; these are regions of high surface roughness, surface defects, and extremely thin or sharp features.

Supplementary **Fig. 32** plots the maximum principal stress with respect to the normalized Gaussian curvature for the stereom, the synthetic foam, and the architected lattice with a similar volume fraction. First, we see that the stereom exhibits the narrowest range of Gaussian curvature, which is in the range of [-100, 20]. In comparison, the range of Gaussian curvatures for the other two structures are [-500, 100] and [-400, 100], respectively. This result is a quantitative description of the fact that stereom has a much smoother surface compared to the reticulated foam and the additively manufactured architected foam. Second, the highest stress point on the stereom surface is found at normalized Gaussian curvature of -26.5, which is much larger than -100 and -307 for the reticulated foam and architected lattice. Based on our discussion on the two scales of surface curvature above, this significant difference further demonstrates that while the highest tensile stress of the stereom is controlled by its branch diameter, the stress concentrations of the synthetic foam and architected lattice are controlled by their local surface and structural defects. This result is consistent with the experimental observation that the stereom has a smooth surface while the synthetic foam and architected lattice have rough surfaces, which unavoidably leads to stress concentration. Third, the stereom also possesses the lowest average level of tensile stress, which is presumably related to its co-continuous morphology. It should also be noted that if we consider the maximum tensile stress of the three structures (marked by arrows in Supplementary **Fig. 32**), the architected foam exhibits the minimum value (21.9 MPa), smaller than that of the stereom (26.5 MPa). However, the architected foam fails catastrophically once the critical strength is reached (Supplementary **Fig. 37**). This is because all the horizontal branches of the architected foam are under significant tension, which fractures easily along the cleavage planes.

In addition to the correlation between the Gaussian curvature and the maximum principal stress discussed above, we further plot stress as functions of the two principal curvatures in Supplementary **Fig. 33c**. It is found that the high-stress regions are scattered in the interfacial shape distribution plots, especially for the synthetic foam. A comparison of the distribution of stress (Supplementary **Fig. 33a**) and surface curvature (Supplementary **Fig. 33b**) shows no definite correlation between high curvature ($\kappa_1 S_v$) and high-stress regions. This is straightforward to

understand, as surface curvature is not the only factor that controls the stress concentration. Many other parameters, including branch orientation, connectivity, and morphologies of adjacent branches all contribute to the stress distribution. As such, large curvature does not always cause stress concentration, although it is positively correlated with high stress. Impressively, the stereom of sea urchin is able to harness curvature to reduce stress concentration synergistically with other factors like structure randomness, varying branch orientation, and low node connectivity.

Supplementary note 7

On the effect of defect and branch size on the bulk strength of biogenic calcite

The critical size a_{c} of the biogenic calcite can be estimated from the correlation[24] between the fracture strength σ_s and fracture toughness K_{IC} in Equation-3,

$$\sigma_s = YK_{\mathrm{IC}}/\sqrt{\pi a_{\mathrm{c}}} \quad (3)$$

Here, we assume an almond-shaped surface crack (crack depth less than 30-50% of the branch diameter) in a branch loaded in tension or bending. The non-dimensional parameter Y is a constant close to unity[25]. As we have discussed in Supplementary Note 4, the bulk strength of biogenic calcite is estimated in the range of 100 to 450 MPa. The fracture toughness K_{IC} of calcite is approximately 0.2 MPa·$m^{0.5}$[26-28]. Using Equation-3, the defect sizes that correspond to the strength of 100 MPa and 450 MPa can then be estimated as 1.3 µm and 80 nm, respectively. It is found that these estimated defect sizes (1.3 µm and 80 nm) are orders of magnitude smaller the branch sizes (~20 µm) of the stereom, which suggests that the material strength of the biogenic calcite is not controlled by the branch size. In other words, there is no direct correlation between the size of the branch and the strength of the material (i.e., no size-strengthening effect from the branch size). This is different from many architected micro-lattices, whose defect size is directly constrained by their feature sizes, thus contributing to their "size effect"-induced strengthening[11,12,13]. In biogenic calcite, intracrystalline organic inclusions are present within the calcite matrix and can be considered as internal defects[8]. Previous TEM analysis has shown that the size of these intracrystalline organic inclusions ranges from 10-20 nm[8]. These results suggest that the material strength of biogenic calcite is not controlled by the branch size but possibly the intracrystalline organic inclusions. In addition, the strength value of 450 MPa which corresponds to a defect size of 80 nm is a better estimation of the tensile strength of biogenic calcite in stereom.

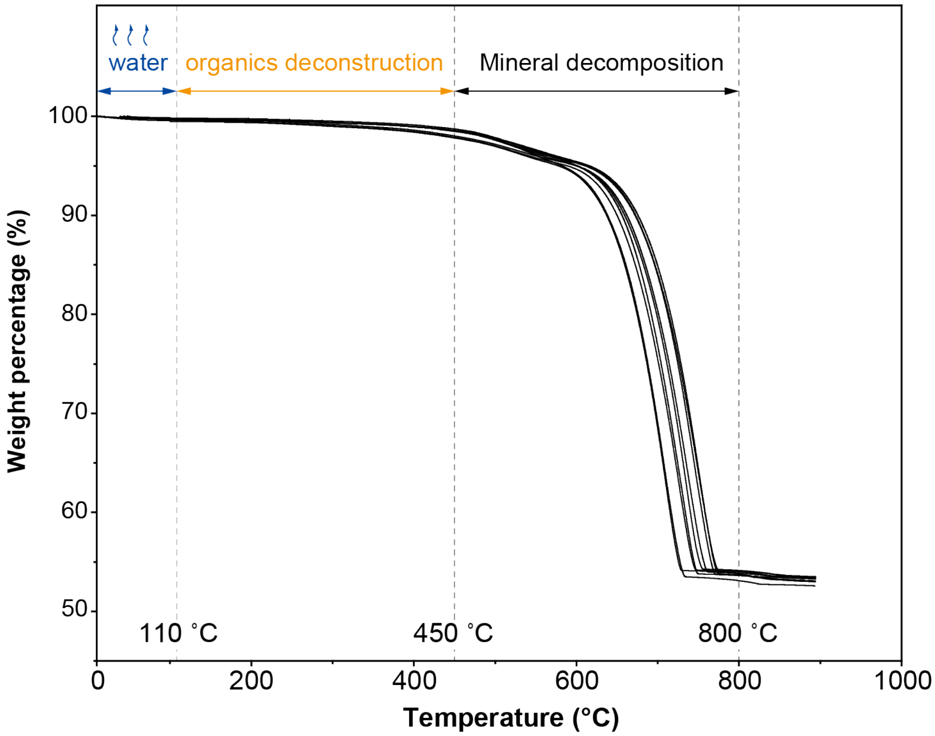

Supplementary Fig. 1. TGA measurements of stereom samples. The weight loss between temperatures 110 ℃ and 450 ℃ is used to estimate the organic content[1].

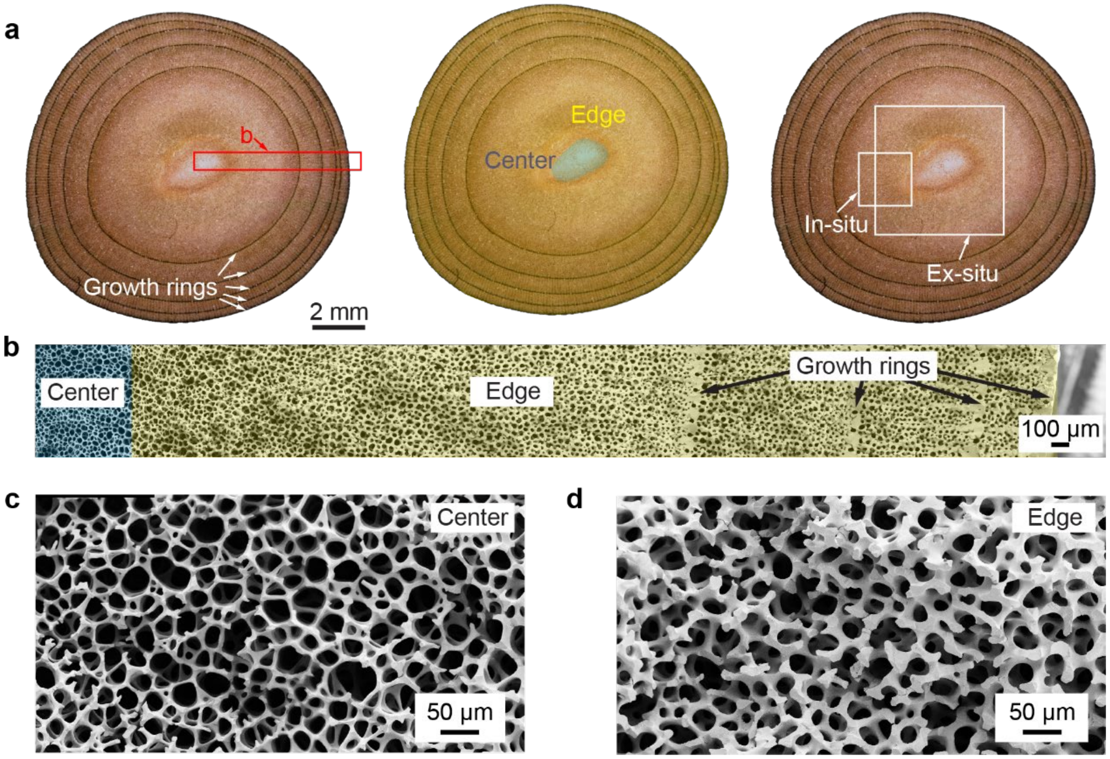

Supplementary Fig. 2. The structural gradient of the cellular structure in sea urchin spines. a, An optical image of the transverse cross-section of a spine. The red boxed region in the left panel indicates the location for the SEM image in (**b**). The blue- and yellow-shaded regions indicate the center and edge regions. The white boxes in the right panel indicate the locations where the volumes were extracted for in-situ and ex-situ mechanical testing. **c**,**d**, High-magnification representative SEM images acquired in the center and edge regions, respectively.

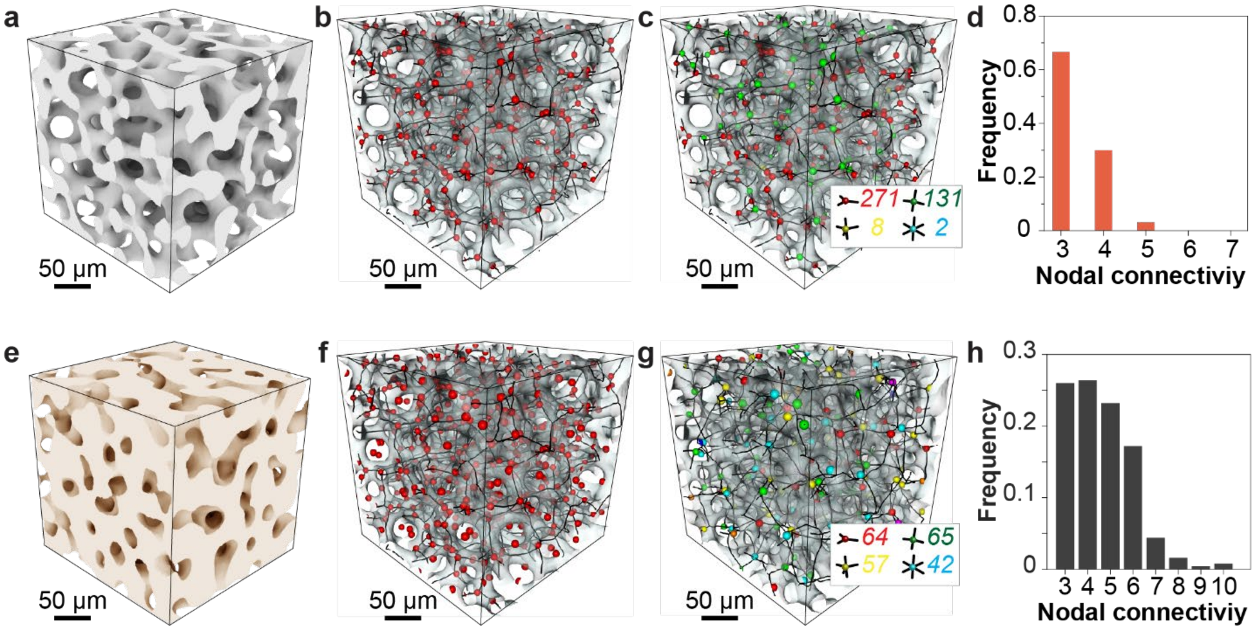

Supplementary Fig. 3. Quantitative cellular network analysis of nodes in (a-d) stereom and (e-h) the inverse structure. (**a,e**) 3D rendering, (**b,f**) the corresponding skeletonized network, (**c,g**) skeletonized network with nodal connectivity indicated with colored nodes and (**d,h**) distribution of nodal connectivity in the volumes.

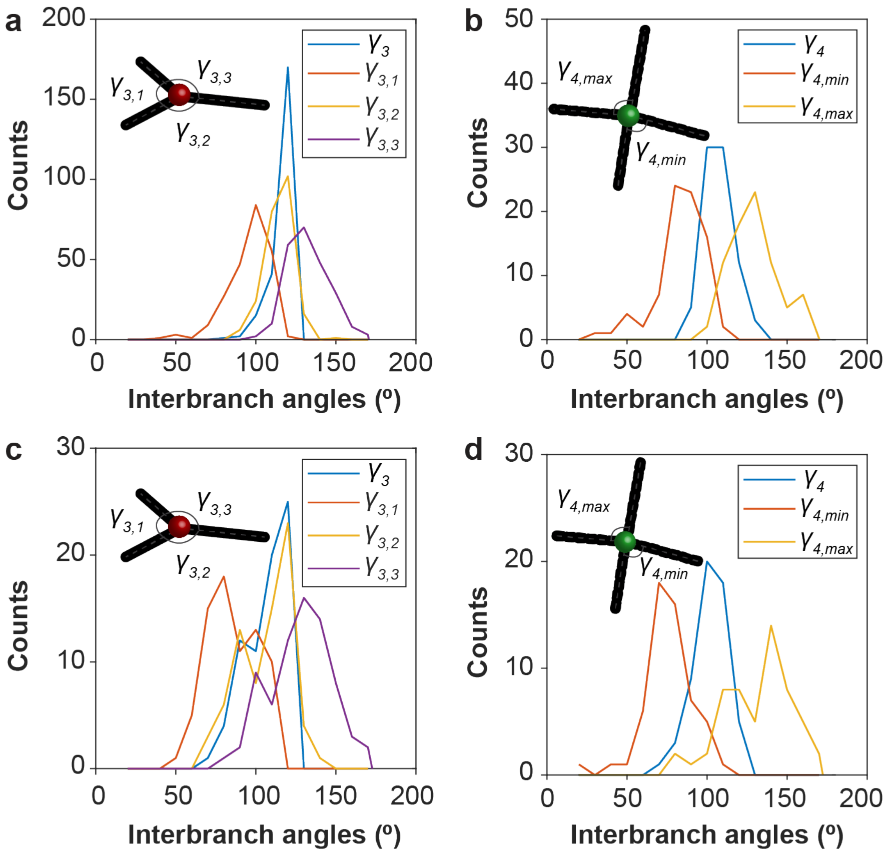

Supplementary Fig. 4. Quantitative analysis of inter-branch angles in the stereom and the inverse structure. a,c, Distribution of individual ($\gamma_{3,1} < \gamma_{3,2} < \gamma_{3,3}$) and mean ($\gamma_3$) inter-branch angles for three-branch nodes and in (**a**) the stereom and (**c**) the inverse structure. **b,d**, Distribution of inter-branch angles ($\gamma_{4,min}$, $\gamma_{4,max}$, and mean value γ_4) for four-branched nodes in (**b**) the stereom and (**d**) the inverse structure.

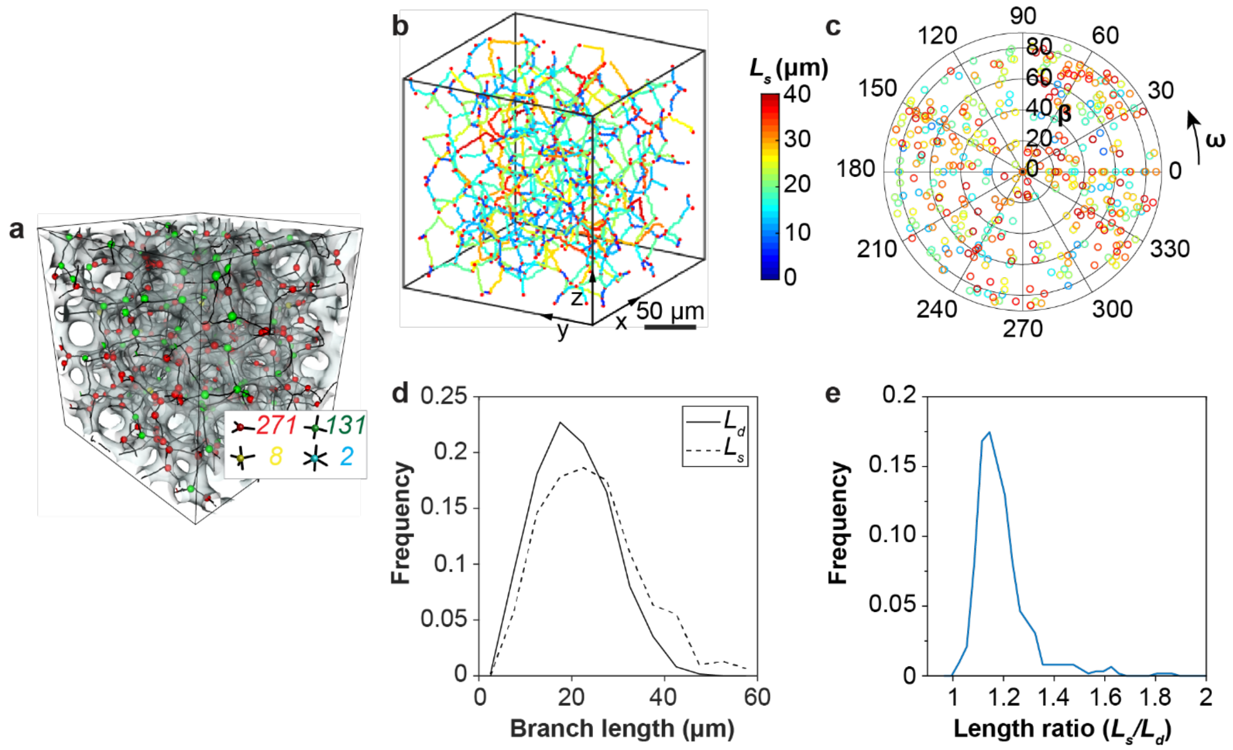

Supplementary Fig. 5. Quantitative analysis of branch length and orientations in stereom. a, Skeletonized network with nodal connectivity indicated with colored nodes. **b**, Network representation of branch length L_s distribution. **c**, Correlation of branch orientation (ω, β) and length (L_s). **d**, Distribution of branch length L_s and branch distance L_d. **e**, Distribution of length ratio (L_s/L_d).

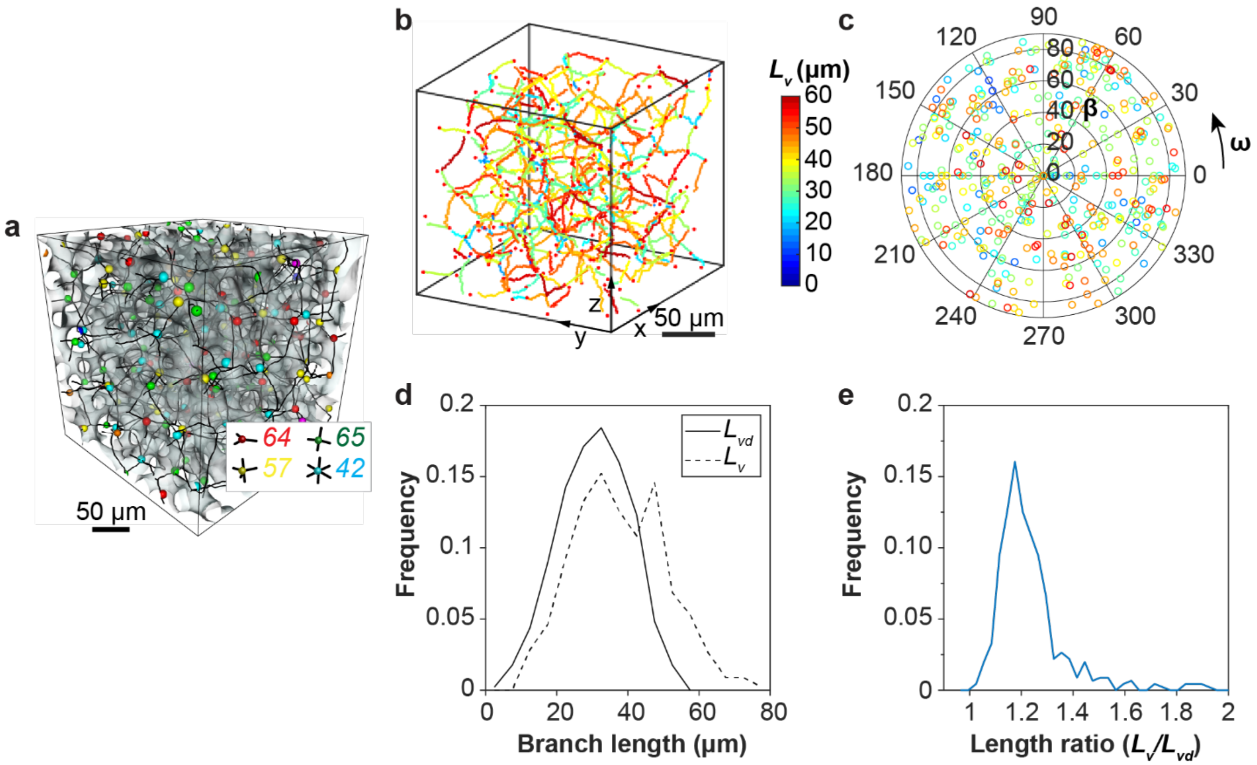

Supplementary Fig. 6. Quantitative analysis of branch length and orientations in the inverse structure. **a**, Skeletonized network with node types indicated with colored nodes. **b**, Network representation of branch length (L_v) distribution. **c**, Correlation of branch orientation (ω, β) and length (L_v). **d**, Distribution of branch length L_v and branch distance L_{vd}. **e**, Distribution of length ratio (L_v/L_{vd}).

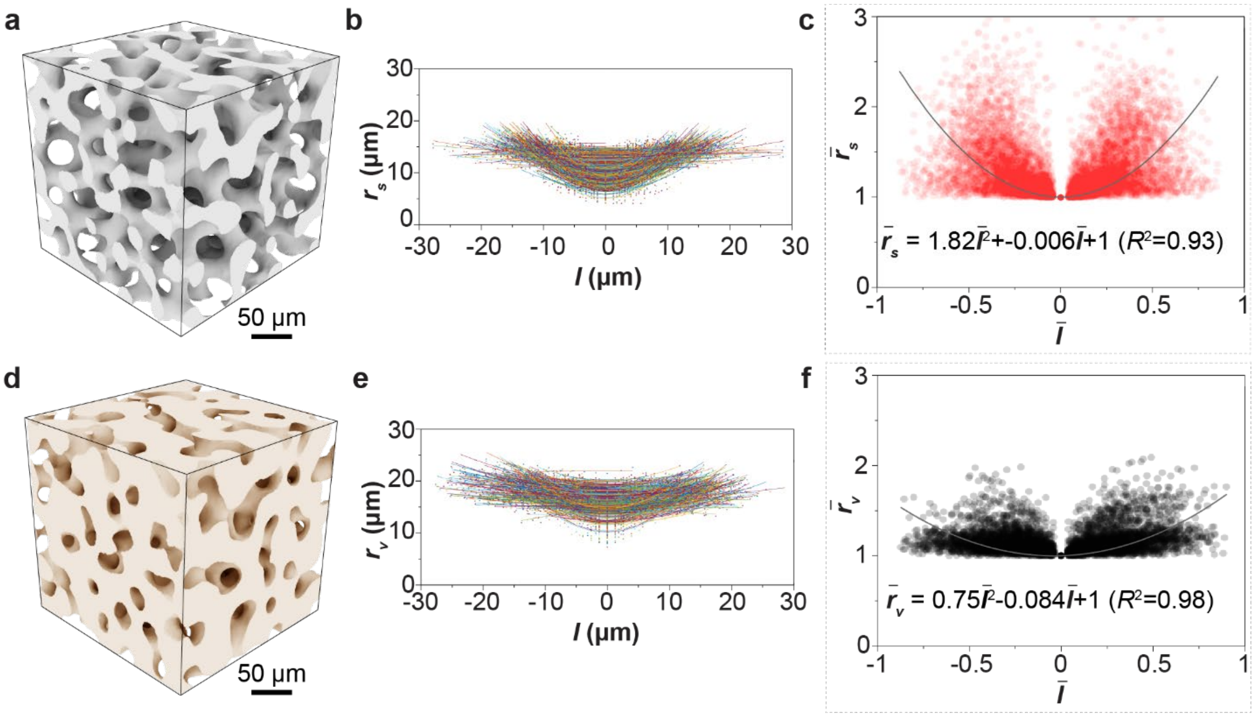

Supplementary Fig. 7. Quantitative analysis of the branch profiles in (a-c) stereom and (d-f) the inverse structure. **a**,**d**, 3D volume rendering. **b**,**e**, Measurements of local branch radius and the fitting results for individual branches in this volume. **c**,**f**, Normalized branch profiles of all branches and the fitting result (grey line). For individual branches in the stereom and in the void space, $r_s(l)$ and $r_v(l)$ are the radius of an equivalent circle with the same area of the intersecting profile between the cross-sectional planes perpendicular to the branch at the sampling point and branch surface at a given location of l. The radius $r_s(l)$ and $r_v(l)$ are normalized by the thinnest radius r_{so} and r_{vo}, respectively. The location l is normalized by branch length L_s and L_v, respectively.

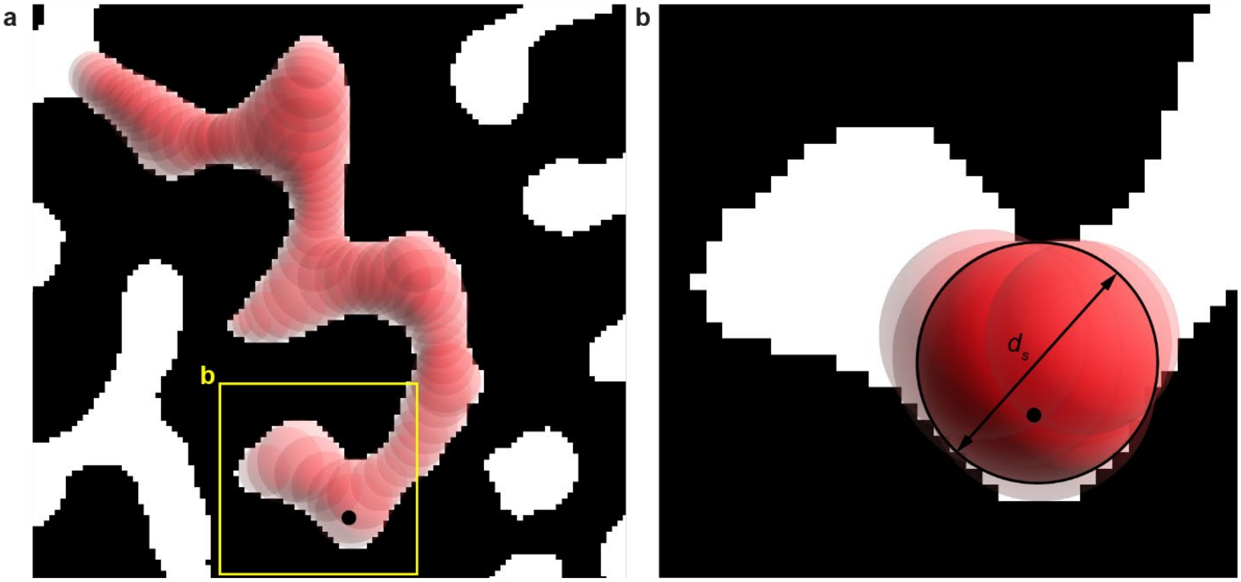

Supplementary Fig. 8. The schematic of local thickness d_s. a, The solid with spheres fully inscribed in the volume. The structure thickness d_s of the local voxel (black dot) is determined by the maximal sphere fully inscribed in the volume, as shown in (**b**). The white region represents the stereom and the black region represents the void structure.

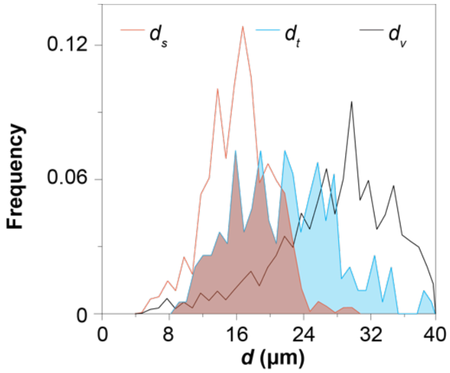

Supplementary Fig. 9. The schematic of the overlap between the probability distributions of the branch thickness (d_s) and the throat size (d_t), where the overlap corresponds to the area of the red shaded region divided by the area of under the curve of d_t.

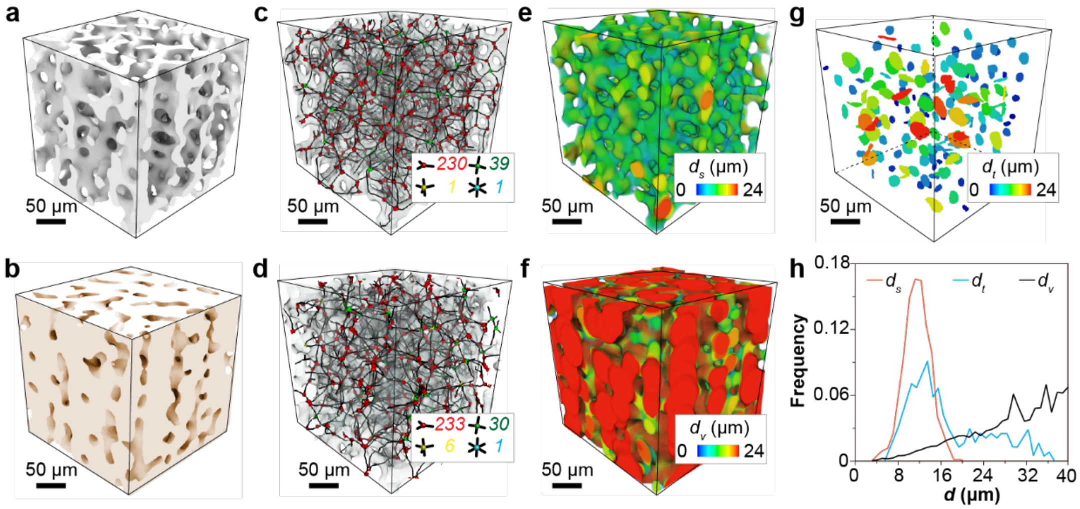

Supplementary Fig. 10. The bicontinuous stereom structure with a relative density of 27%. **a,b**, μ-CT reconstructions of stereom (**a**) and the corresponding void structure (**b**). **c,d,** 3D cellular network of stereom and the corresponding void structure with node types colored by their connectivities. d_s, d_v, and d_t represent the thicknesses (diameters) of stereom, void structure, and throats, respectively. **e,f,** The thickness distributions of stereom (d_s) and the corresponding void structure (d_v). **g**, 3D rendering of small throats ($d_t < 24\ \mu m$) for volume (**a**). **h**, Distribution of d_s, d_v, and d_t, respectively.

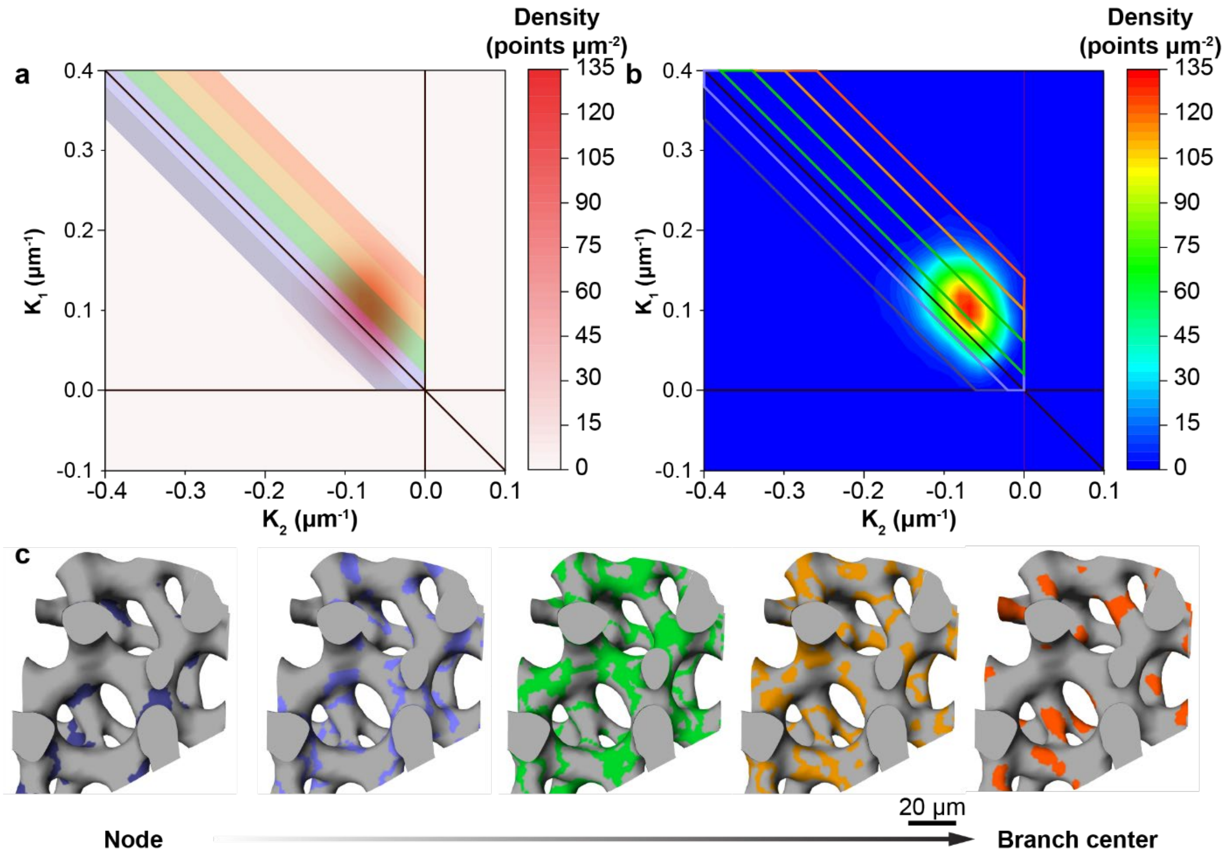

Supplementary Fig. 11. Curvature analysis result of stereom surface. a,b, Interfacial shape distribution (ISD) plots marked by different color palettes. **c**, Corresponding 3D plots of a small volume, where different color represents regions with different mean curvature H ranges indicated in the (**a,b**) ISD plots. The dark blue, purple, green, light orange and orange regions correspond to surface with -0.03 $\mu m^{-1} < H <$ -0.01 μm^{-1}, -0.01 $\mu m^{-1} < H <$ 0.01 μm^{-1}, 0.01 $\mu m^{-1} < H <$ 0.03 μm^{-1}, 0.03 $\mu m^{-1} < H <$ 0.05 μm^{-1}, and 0.05 $\mu m^{-1} < H <$ 0.07 μm^{-1}, respectively.

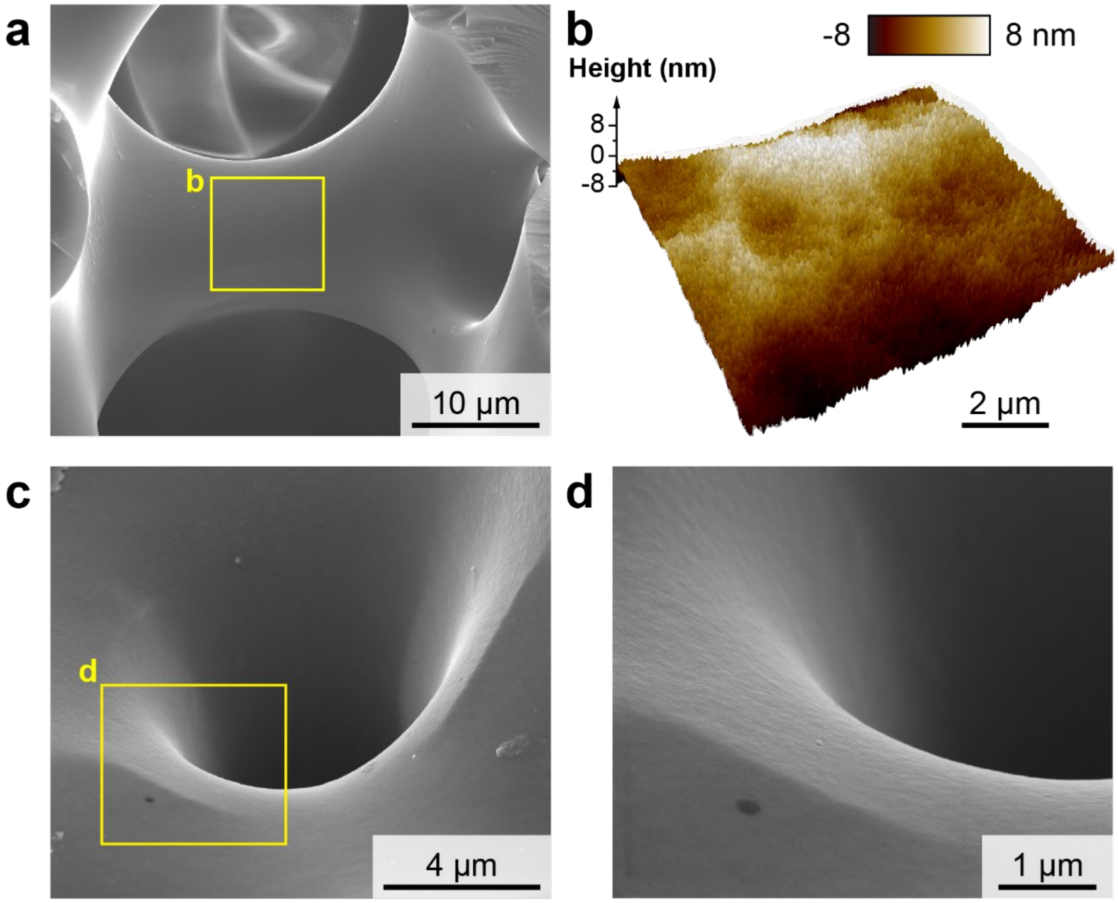

Supplementary Fig. 12. Smooth surface on stereom. a, An SEM image of a representative branch in stereom. **b,** A typical AFM height image acquired from the central region of a branch, demonstrating its nanoscopic surface roughness. **c,d,** SEM images showing the smooth surface of the stereom structure.

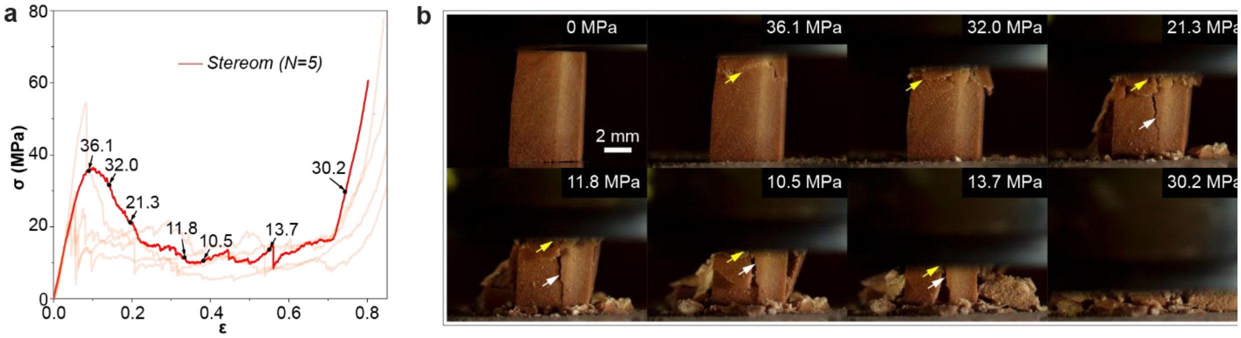

Supplementary Fig. 13. Uniaxial compressive responses of the sea urchin stereom with height to width ratio ~1.5. Stress-strain curves (**a**) and the snapshots (**b**) of corresponding deformation stages for samples from sea urchin stereom. The yellow and white arrows indicate the horizontal damage bands and the vertical spallation, respectively.

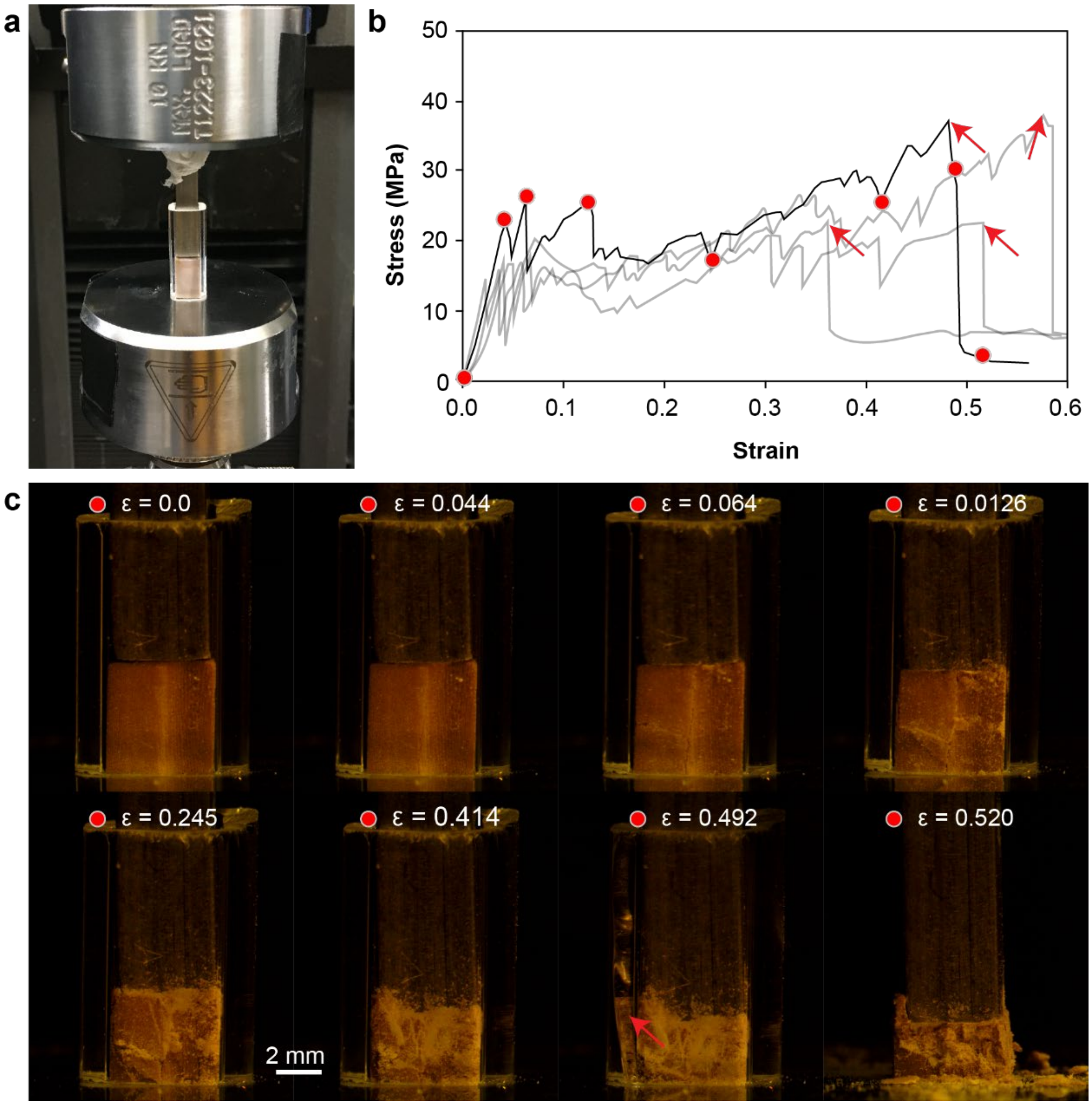

Supplementary Fig. 14. Constrained compression tests of stereom samples. **a**, Experiment setup of the compression test. **b**, Stress-strain curves of the compression tests. **c**, Snapshots of deformation stages corresponding to a representative curve in (**b**). The red arrows indicate the break of the glass tube.

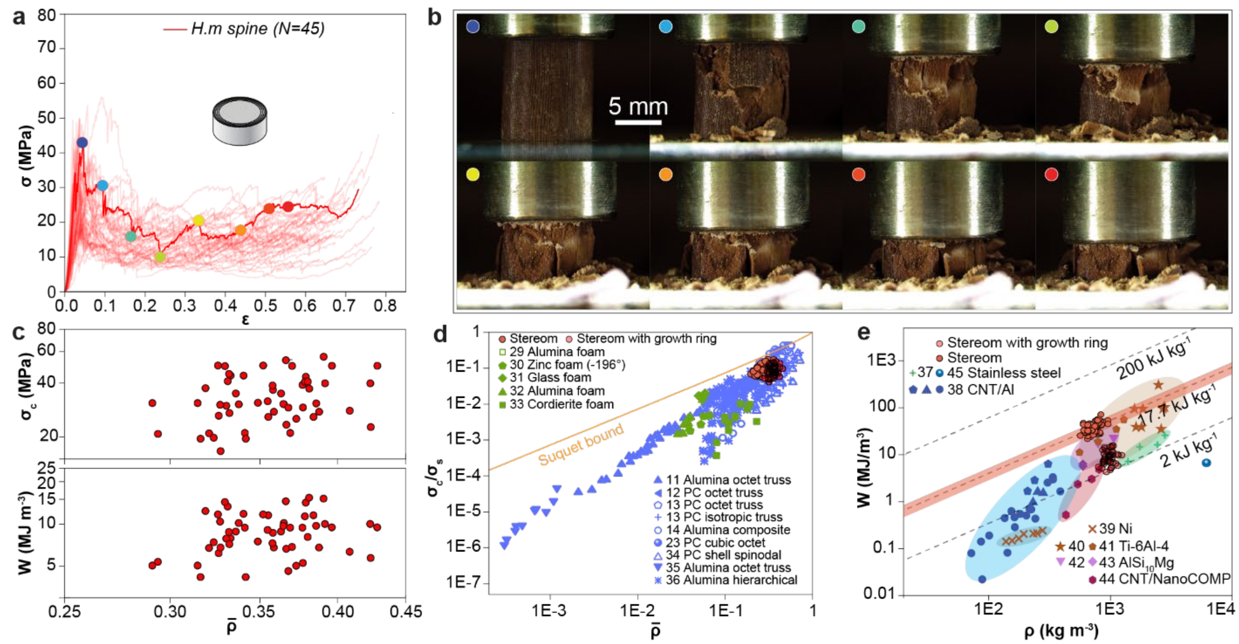

Supplementary Fig. 15. Mechanical properties of the echinoderm stereom with growth rings from *H. mamillatus* spines. a, Stress-strain curves for 45 compression tests. **b,** Snapshots of deformation stages corresponding to a representative curve in (**a**). **c**, Distributions of compressive strength, σ_c, and energy absorption capacity, W, versus relative density, $\bar{\rho}$. **d**, Relative compressive strength, σ_c/σ_s, versus $\bar{\rho}$ (σ_s: fracture strength of solid struts), in comparison to the traditionally[29-33] and additively[11-14,23,34-36] manufactured ceramic foams. **e**, The energy absorption capacity, W, versus density, ρ, in comparison to synthetic cellular solids[37-45]. The dashed lines represent different W/ρ values, and the shaded area highlights the standard deviation of W/ρ for stereom.

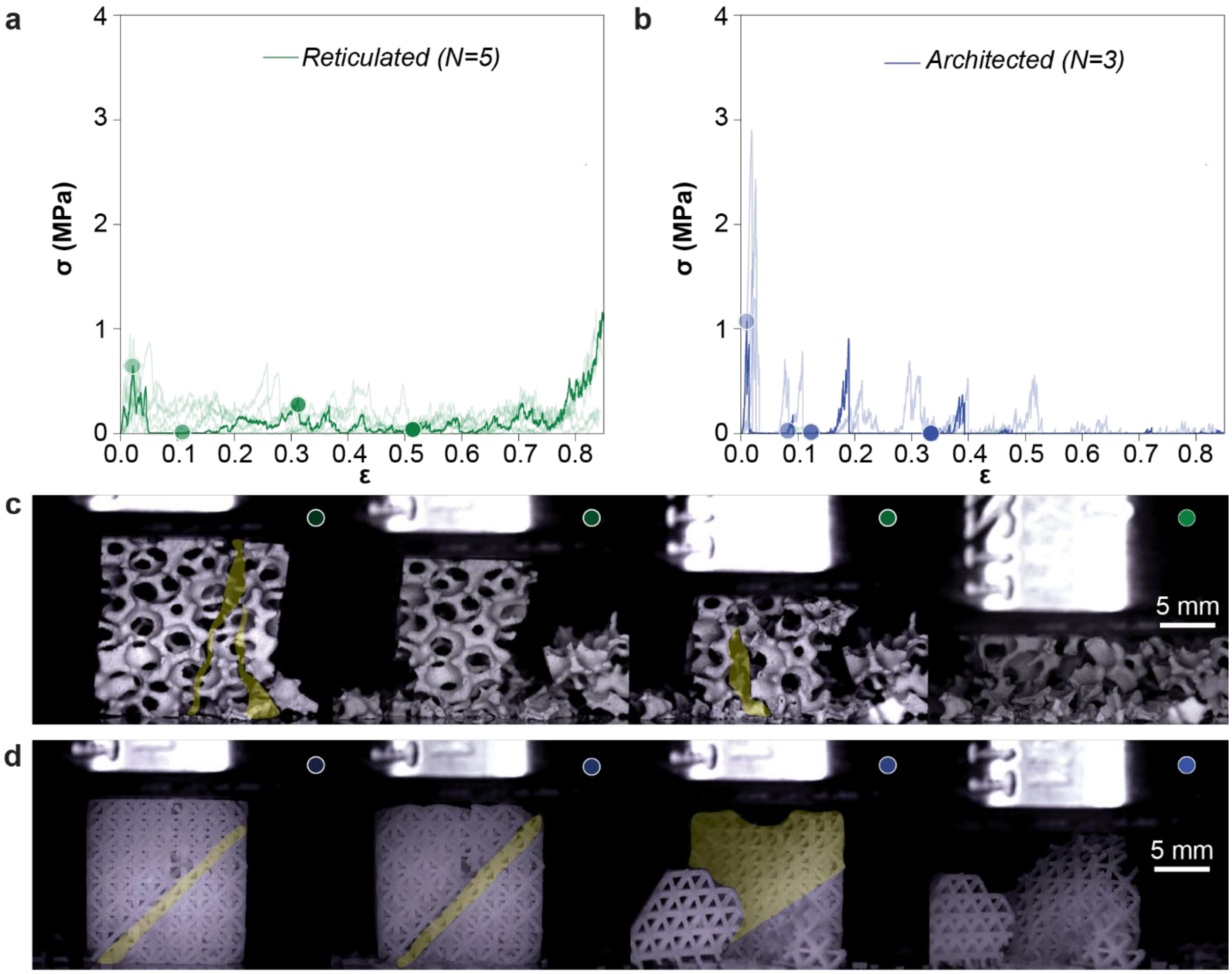

Supplementary Fig. 16. Uniaxial compressive responses of the engineering ceramic foams. Stress-strain curves and the snapshots of corresponding deformation stages for samples from the alumina-based reticulated foam (**a,c**) and silica-based additively manufactured octet truss (**b,d**). The fracture plane of the octet truss is shaded in yellow.

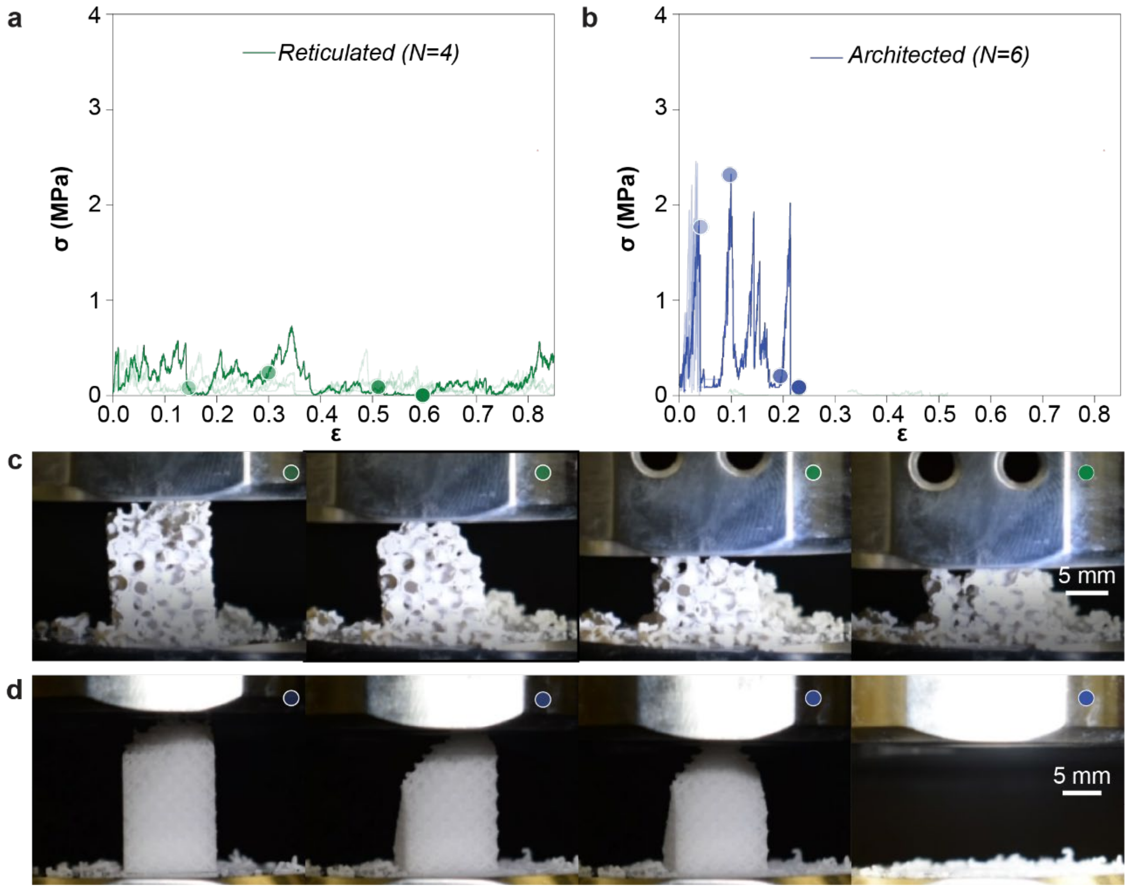

Supplementary Fig. 17. Uniaxial compressive responses of the engineering ceramic foam with height to width ratio ~1.5. Stress-strain curves and the snapshots of corresponding deformation stages for samples from (**a,c**) the alumina-based reticulated foam and (**b,d**) silica-based additively manufactured octet truss.

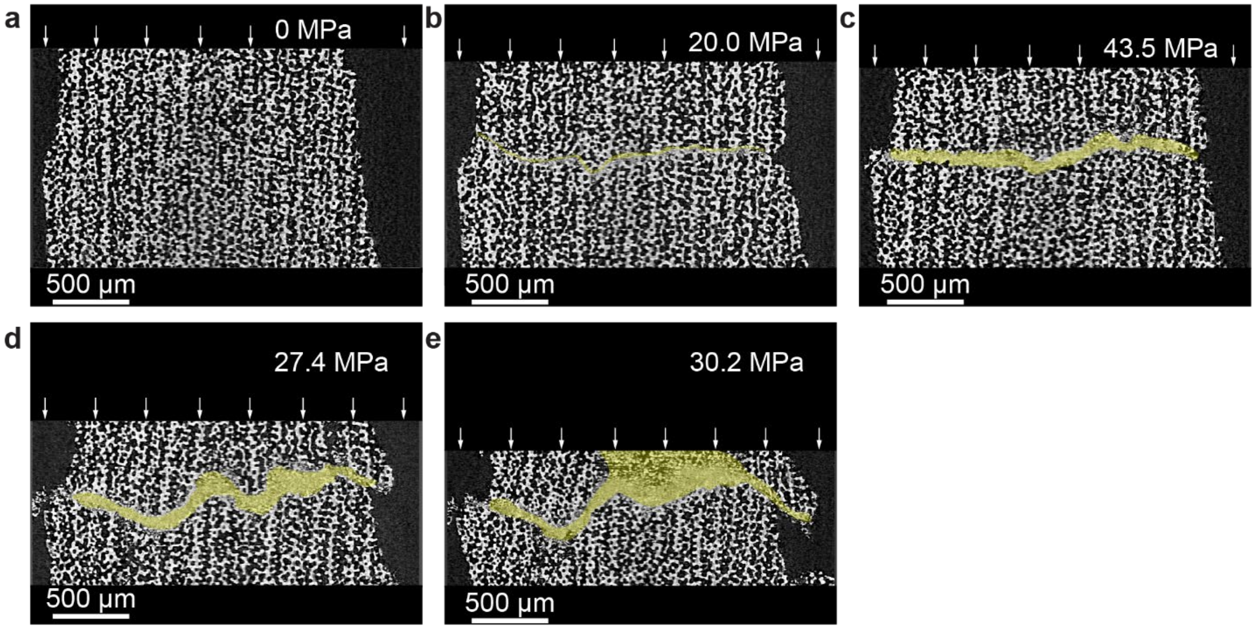

Supplementary Fig. 18. Vertical slices showing the damaged stereom at applied stress (a) $\sigma = 0\ MPa$, (b) $\sigma = 20.0\ MPa$, (c) $\sigma = 43.5\ MPa$, (d) $\sigma = 27.4\ MPa$, and (e) $\sigma = 30.2\ MPa$. The damage band is shaded in yellow.

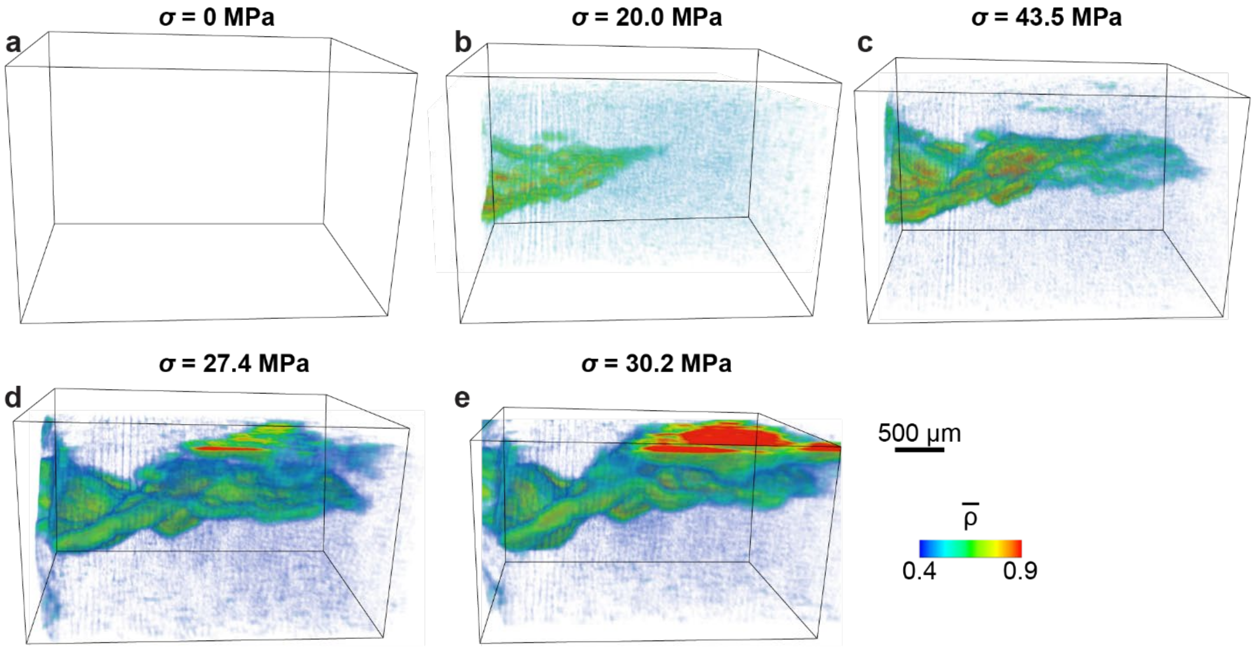

Supplementary Fig. 19. The local relative density ($\overline{\rho}$) map for (a-e) different deformation stages of stereom upon compression obtained from the synchrotron in-situ measurements. All panels share the same scale bar and color bar.

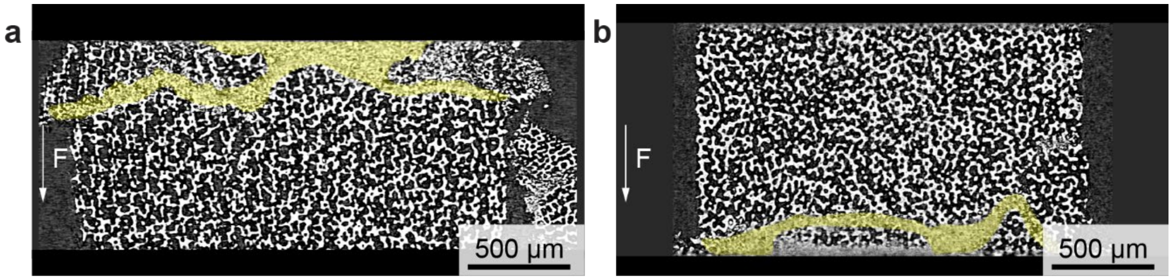

Supplementary Fig. 20. Examples of damage bands formed at different locations of the stereom. a,b, The damage band formed at (**a**) the top and (**b**) the bottom of the specimen upon compression. The damage band is shaded in yellow.

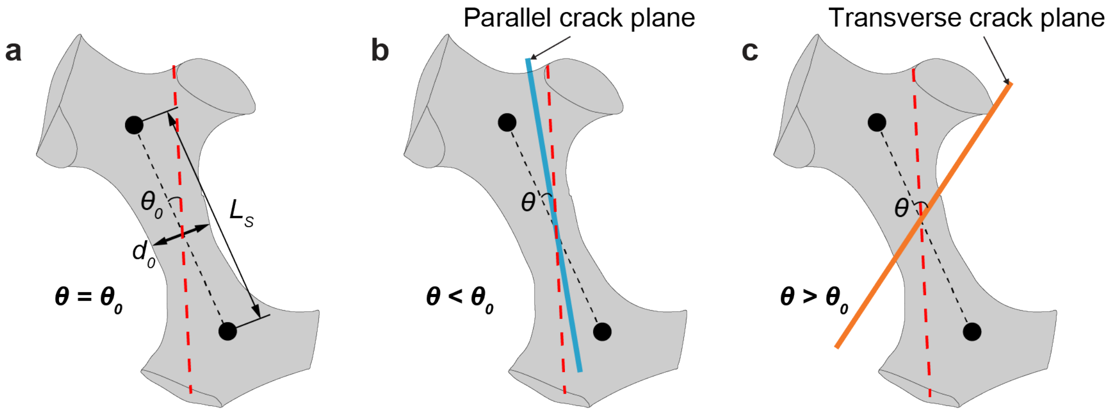

Supplementary Fig. 21. Schematic diagram of crack orientation ($\boldsymbol{\theta}$) with respect to the critical value $\boldsymbol{\theta_0}$. a, The illustration of θ_0, where θ_0 can be calculated as $\theta_0 = atan(d_0/L_s)$. **b**, The parallel cracks (colored in blue) with $\theta < \theta_0$. **c**, The transverse cracks (colored in orange) with $\theta < \theta_0$.

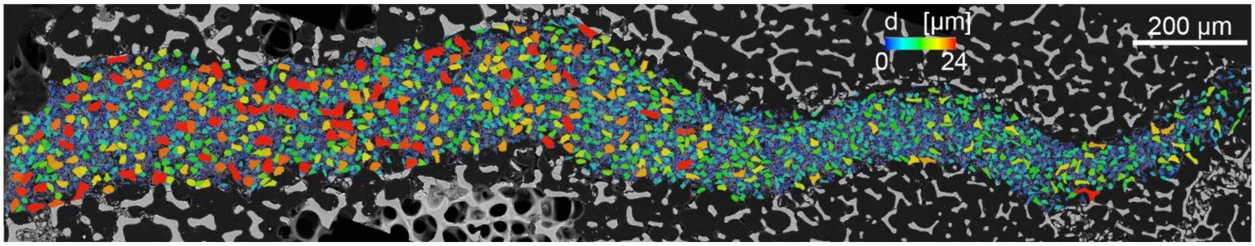

Supplementary Fig. 22. Fragment size distribution in damage bands. The fragments in the damage band are indicated by their equivalent diameter *d*.

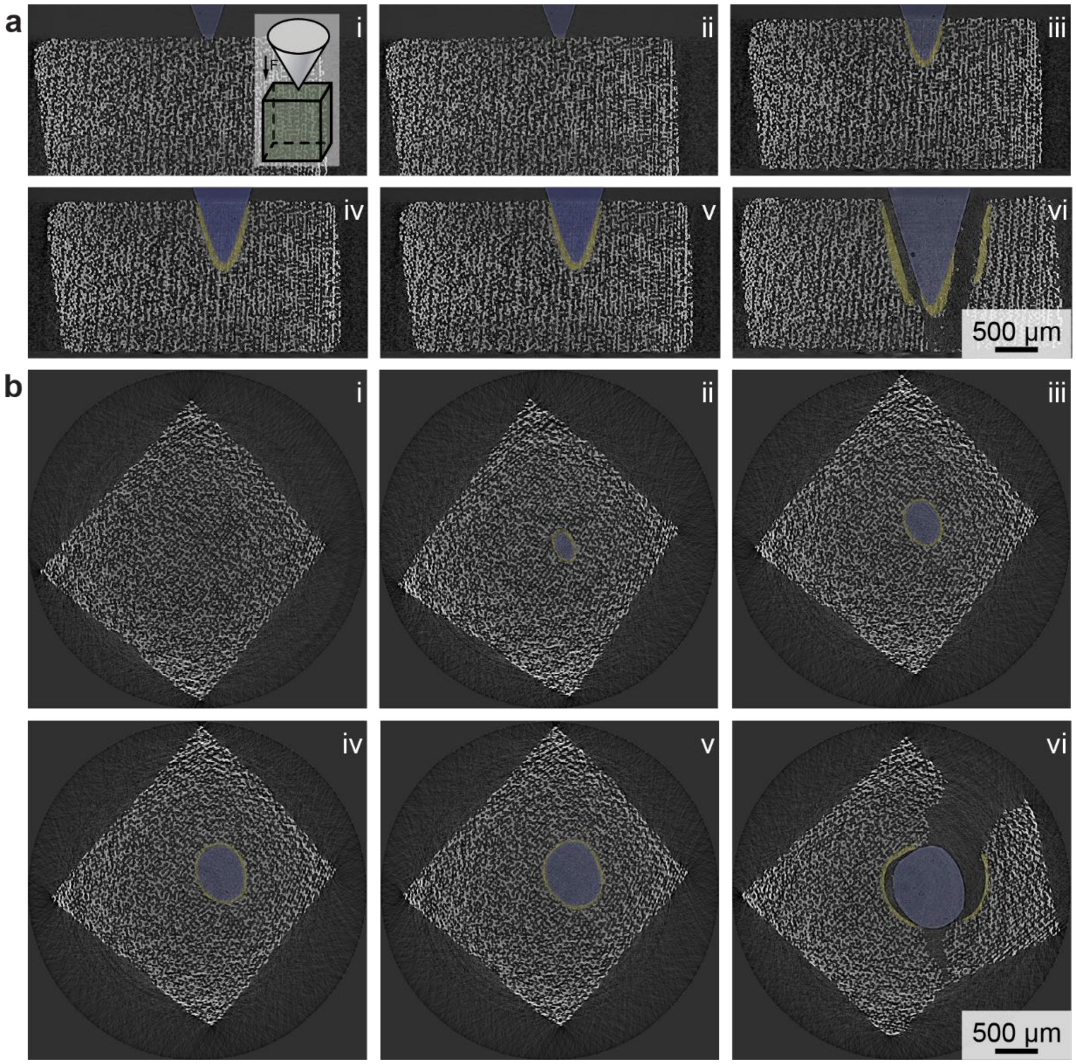

Supplementary Fig. 23. The reconstruction slices showing damaged stereom upon indentation experiments at different deformation stages. (**a**) and (**b**) represent vertical slices and horizontal slices of damaged stereom at six deformation stages (i-vi), respectively. The indenter is shaded in purple, and the damage band is shaded in yellow, respectively.

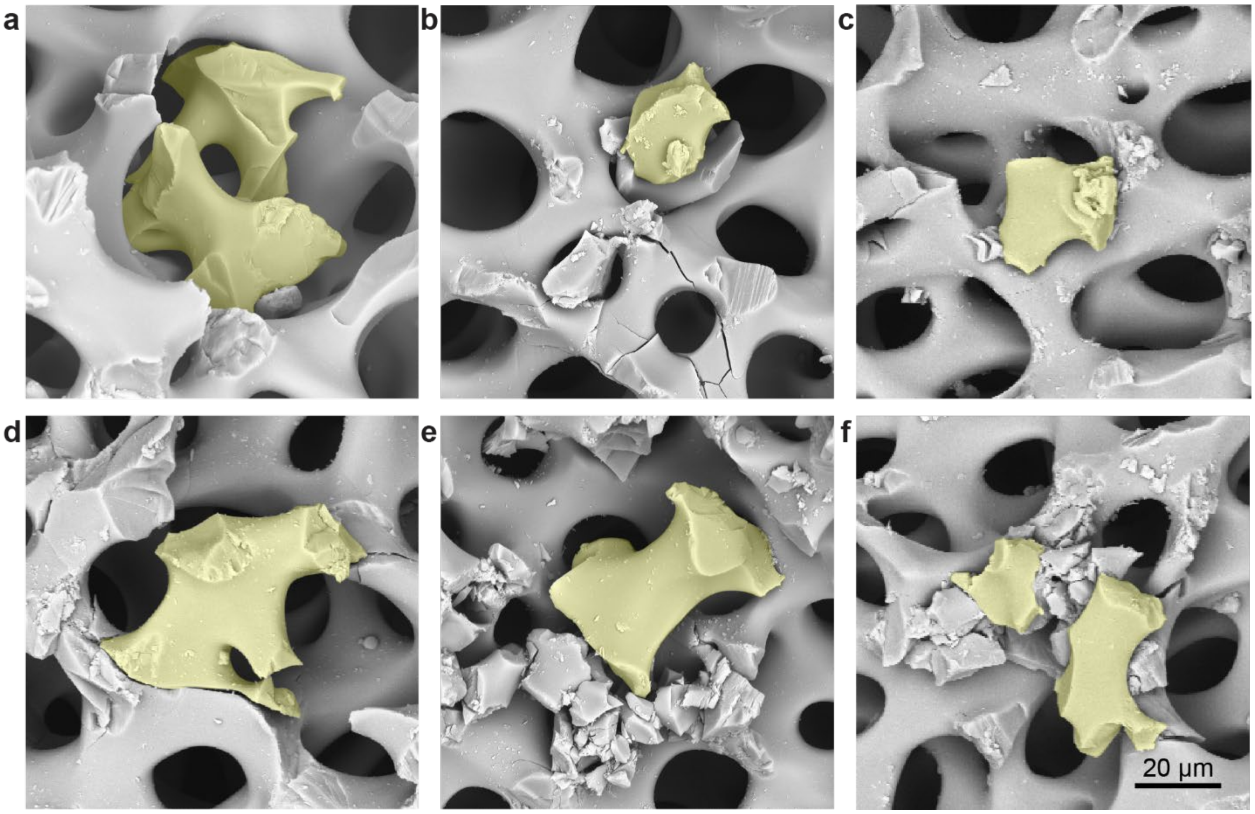

Supplementary Fig. 24. SEM images of the compressive residue showing the jamming of fragments in the stereom. a-f, The jamming of fragments at different locations. The fragments are shaded in yellow, and all panels share the same scale bar.

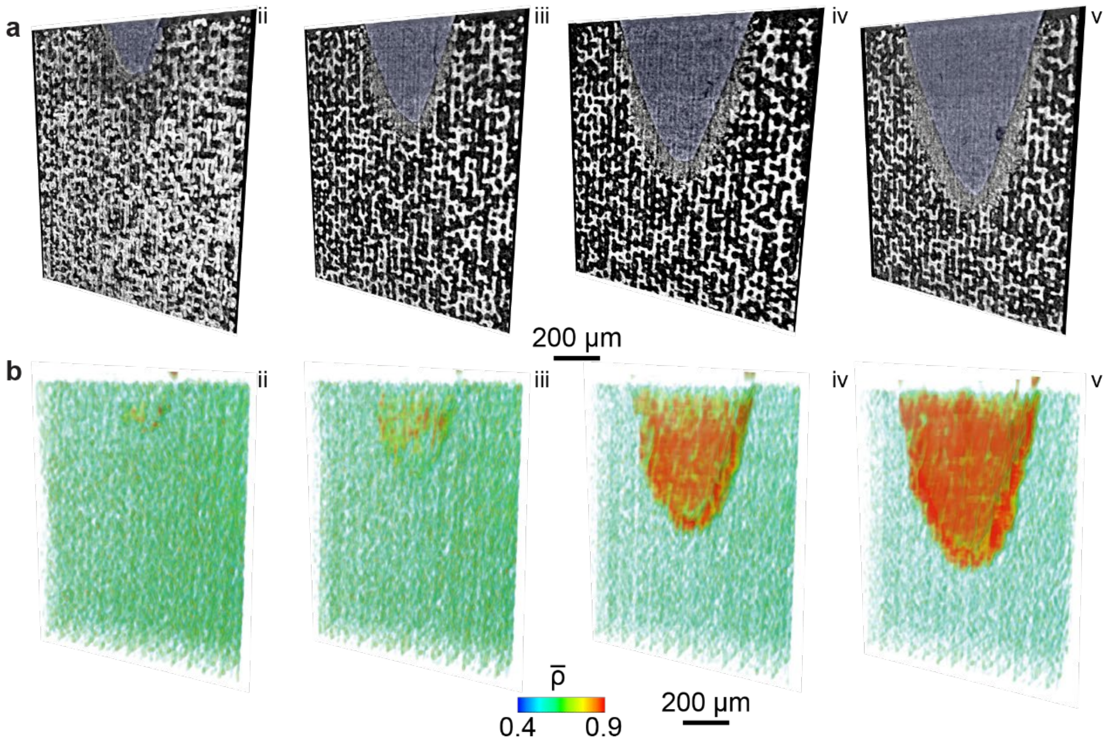

Supplementary Fig. 25. The local relative density ($\overline{\rho}$) distribution of the damaged stereom upon indentation at different deformation stages (i-v). (a) and **(b)** represent vertical reconstruction slices and the local density map for the damaged stereom, respectively.

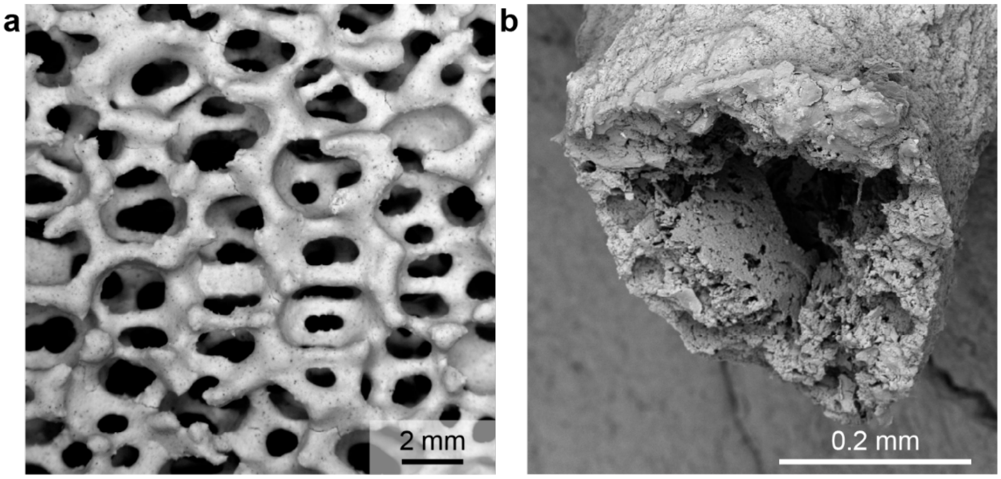

Supplementary Fig. 26. The structure of alumina-based reticulated foam fabricated from the replication process. a, Photograph of the alumina-based reticulated foam. **b**, An SEM image of a fractured branch.

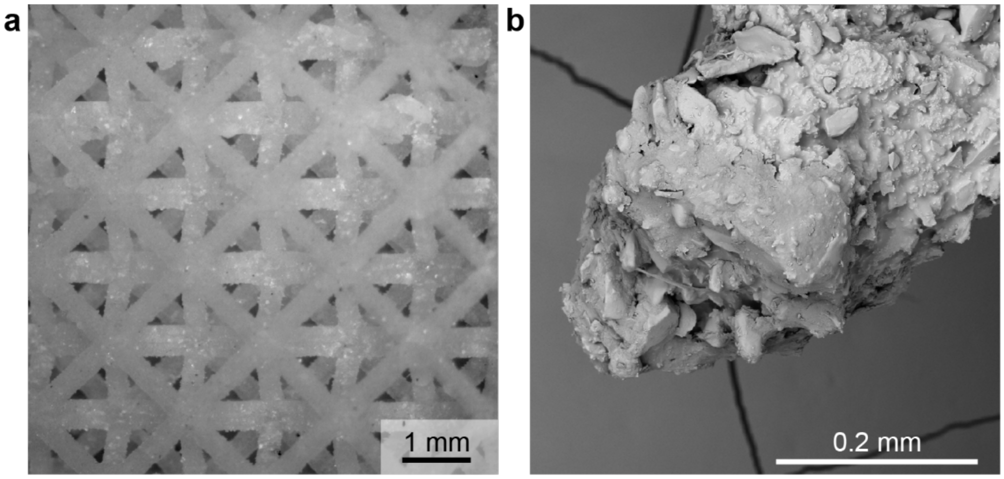

Supplementary Fig. 27. Structure of silica-based octet truss fabricated from additive manufacturing. a, Photograph of the as-fabricated sample. **b**, An SEM image of a fractured branch.

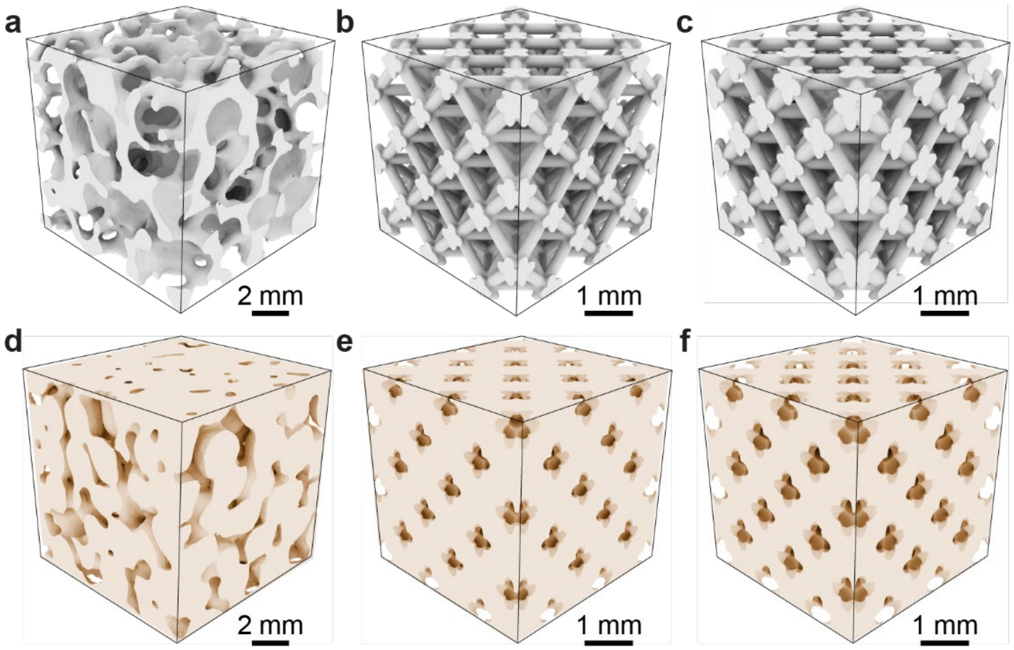

Supplementary Fig. 28. 3D rendering of the reticulated foam, architected foams, and their corresponding inverse structures. (**a**) and (**d**) represent the reticulated foam with $\bar{\rho} = 0.29$ and the inverse structure, respectively. (**b**,**c**) and (**e**,**f**) represent the architected foams with (**b**) $\bar{\rho} = 0.26$ and (**c**) $\bar{\rho} = 0.34$, and (**e**,**f**) the inverse structures, respectively.

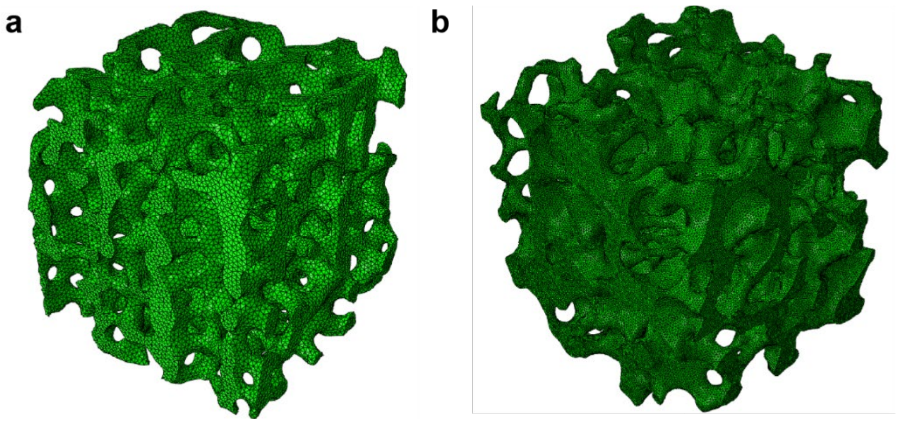

Supplementary Fig. 29. Meshes used in the simulation for the stereom and reticulated foams. **a,b,** the mesh distribution for (**a**) the stereom and (**b**) the reticulated foam. The mesh density is based on the smallest branch size, i.e., 5 elements are used in the thickness direction of the thinnest branches.

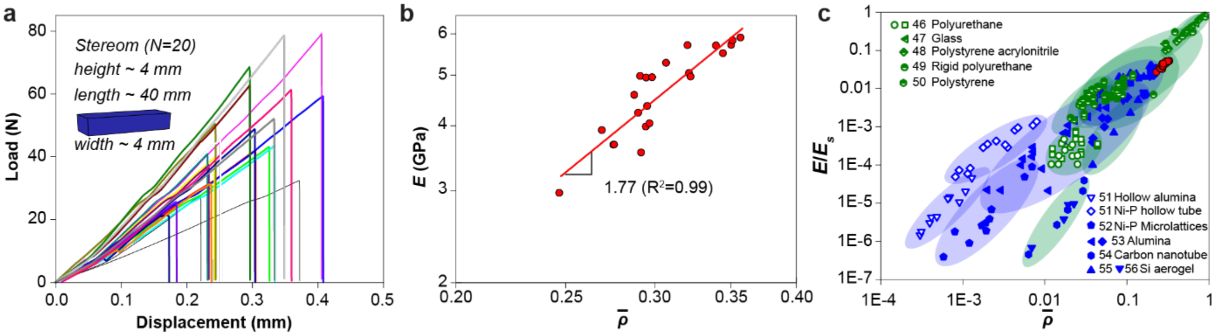

Supplementary Fig. 30. The mechanical properties of stereom measured from three-point bending tests. a, Load-displacement curves for 20 three-point bending tests. The inset box is a schematic of specimen used in tests, with dimensions labeled. **b,** The elastic modulus, E, versus relative density $\bar{\rho}$. **c**, Relative modulus, E/E_s, versus $\bar{\rho}$ (E_s: modulus of the geological calcite), in comparison to conventionally[46-50] and additively[51-56] manufactured ceramic foams.

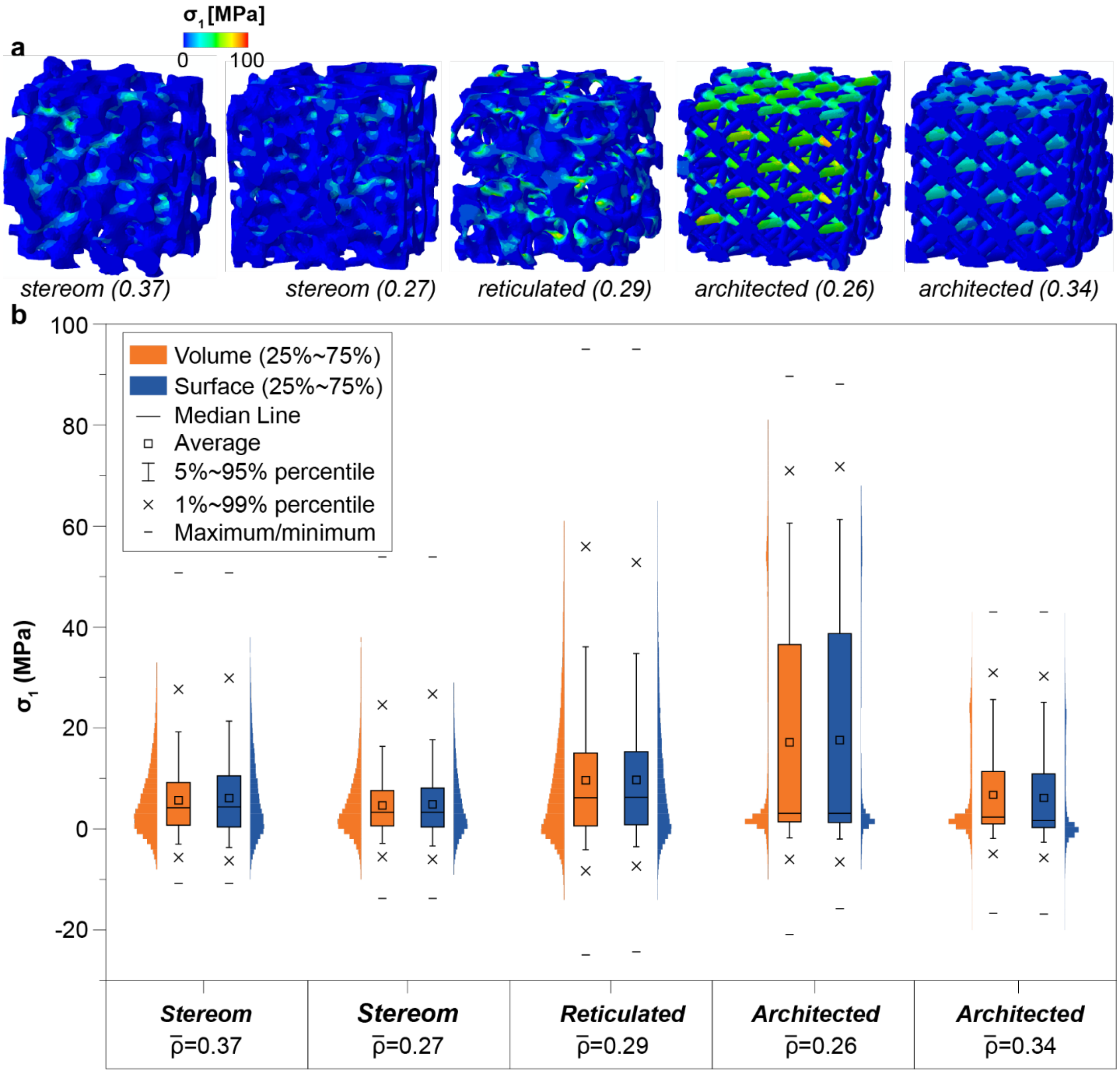

Supplementary Fig. 31. Quantitative analysis of maximum principal stresses of different ceramic foams. a, Contour plots of maximum principal stress distributions on the stereom ($\bar{\rho} = 0.37, 0.27$), reticulated foam ($\bar{\rho} = 0.29$), and the architected foams ($\bar{\rho} = 0.26, 0.34$). **b**, The corresponding statistical results of maximum principal stresses. The orange and blue colors show statistical analysis based on the total volume and only the surface, respectively.

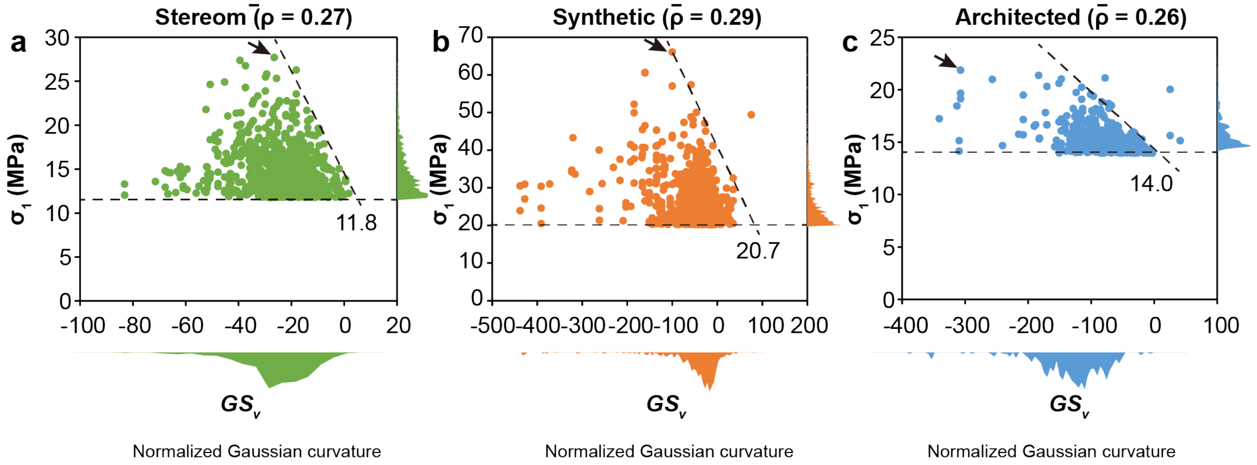

Supplementary Fig. 32. Correlation between the maximum principal stress and the normalized Gaussian curvature for three structures (**a**) stereom ($\bar{\rho}$ = 0.27), (**b**) synthetic foam ($\bar{\rho}$ = 0.29), (**c**) architected foam ($\bar{\rho}$ = 0.26). The Gaussian curvature G is normalized by the characteristic length of the corresponding structure, S_v, which is the sum of average branch diameter and average throat diameter. The arrows mark the data points of highest stress for each structure. The dashed lines are marked to guide the view.

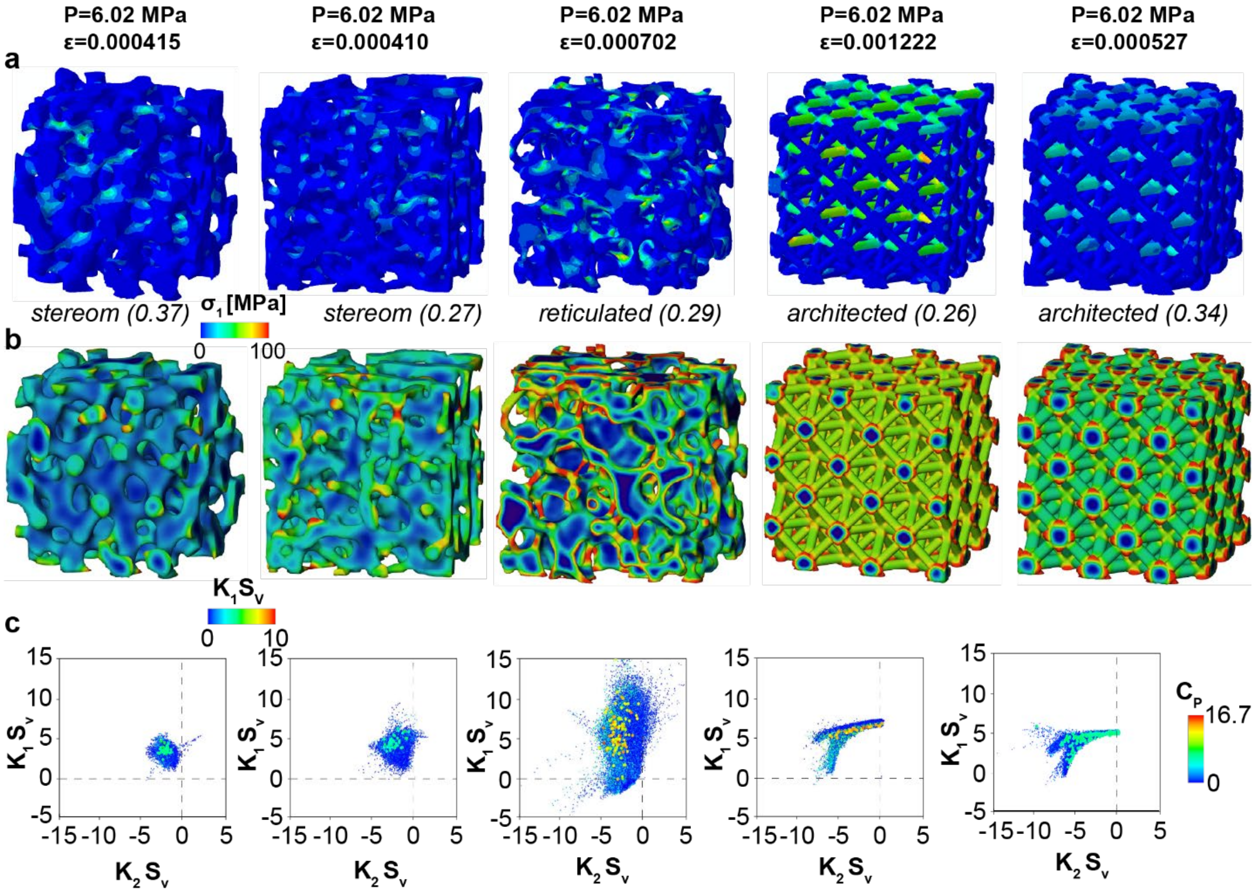

Supplementary Fig. 33. Correlation between the maximum curvature distribution and maximum principal stress of five models under compression at applied stress P = 6.02 MPa. **a**, Maximum principal stress distribution on five models. **b**, Maximum principal curvature distribution. **c**, Scatter plots of normalized principal curvatures, where S_v is the sum of average branch diameter and average throat diameter. for the corresponding structures. The corresponding characteristic lengths S_v equal 38 μm, 31.2 μm, 898 μm, 817 μm, 864 μm for the five models. Each point is colored with the stress concentration factor C_p, which are calculated by the local stress divided by the applied load.

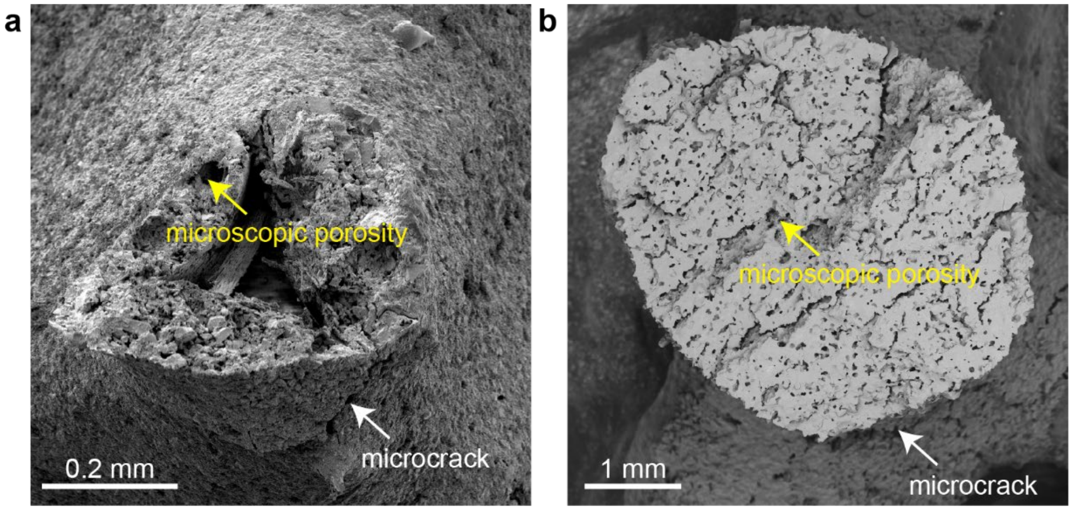

Supplementary Fig. 34. Microscopic defects in (a) the reticulated foam and (b) the architected foam fabricated from the replication process and additive manufacturing, respectively. Two examples of microcracks and microscopic porosity are indicated by white arrows and yellow arrows, respectively.

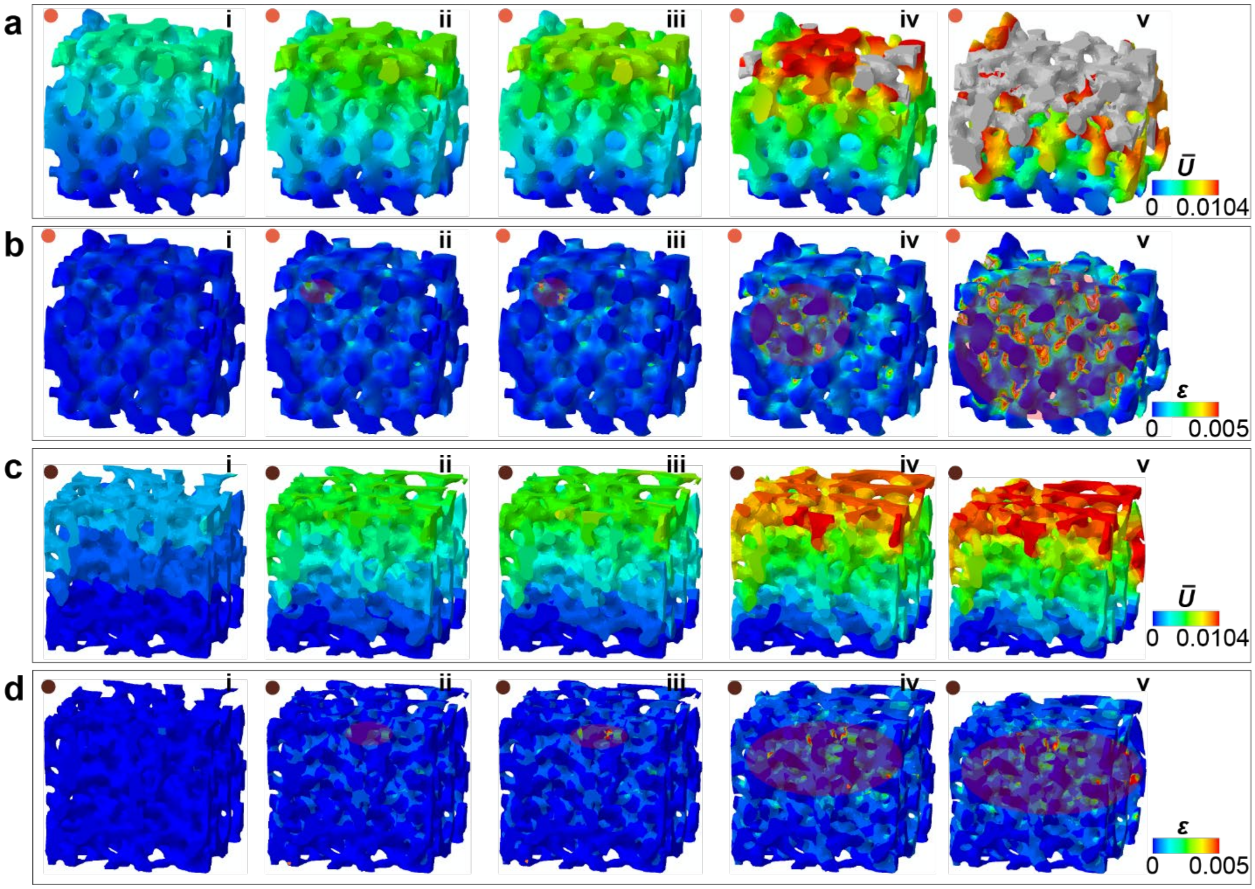

Supplementary Fig. 35. Distributions of normalized displacement $\bar{U}$ and the maximum principal strain ε on the stereom model at different applied strains of (i) 0.0007, (ii) 0.0012, (iii) 0.0013, (iv) 0.0018, (v) 0.0029. (**a,b**) and (**c,d**) represent the distributions on the stereom model with (**a,b**) $\bar{\rho} = 0.37$ and (**c,d**) $\bar{\rho} = 0.27$, respectively. The red shaded regions mark the locations of cracks. The displacement U is normalized by the characteristic length S_v ($\bar{U} = U/S_v$), which is the sum of average branch diameter and average throat diameter, 38 μm and 31.2 μm for stereom with $\bar{\rho} = 0.37$ and 0.27, respectively.

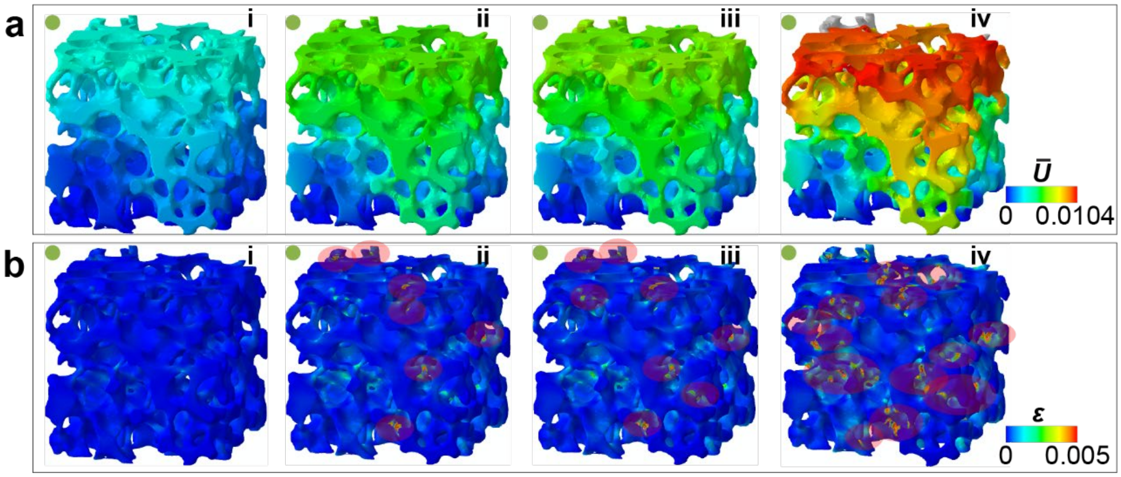

Supplementary Fig. 36. Distributions of normalized displacement $\bar{U}$ and the maximum principal strain ε on the reticulated foam model at different applied strains of (i) 0.0007, (ii) 0.0012, (iii) 0.0013, (iv) 0.0018. a,**b**, distributions of (**a**) the displacement and (**b**) maximum principle strain on the reticulated foam model with $\bar{\rho} = 0.29$, respectively. The red shaded regions mark the locations of cracks. The displacement U is normalized by the characteristic length S_v ($\bar{U} = U/S_v$, 898 μm for the reticulated foam), which is the sum of average branch diameter and average throat diameter.

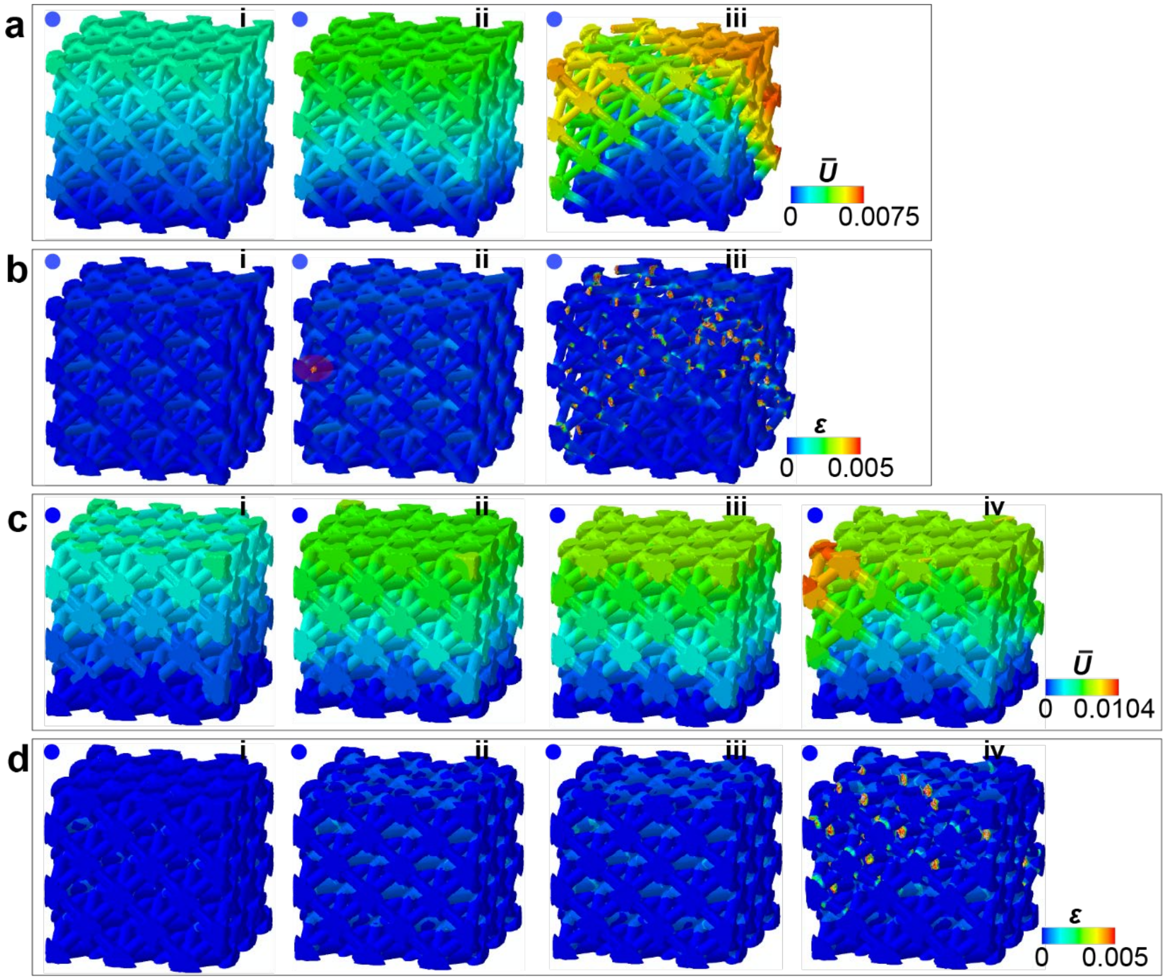

Supplementary Fig. 37. Distributions of normalized displacement $\bar{U}$ and the maximum principal strain ε on the architected foam model at different applied strains of (i) 0.0007, (ii) 0.0012, (iii) 0.0013, (iv) 0.0018. (**a,b**) and (**c,d**) represent the distributions on the architected foam models with (**a,b**) $\bar{\rho} = 0.34$ and (**c,d**) $\bar{\rho} = 0.26$, respectively. The red shaded region marks the locations of cracks. The displacement U is normalized by the characteristic length S_v ($\bar{U} = U/S_v$), which is the sum of average branch diameter and average throat diameter, 864 μm and 817 μm for architected foams with $\bar{\rho} = 0.34$ and 0.26, respectively.

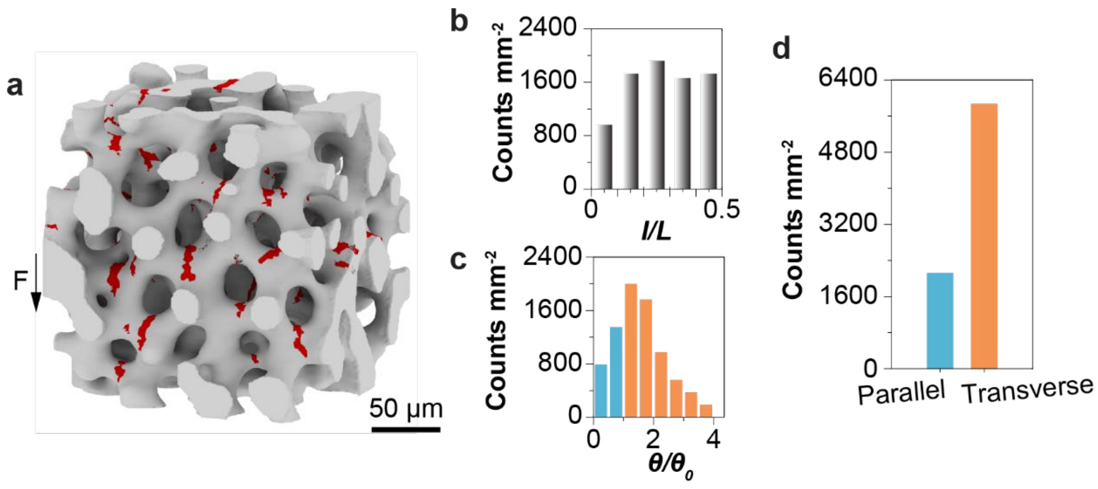

Supplementary Fig. 38. Simulated distribution of microcracks in stereom. a, 3D rendering of microcracks in stereom. **b,c,** The measured location l_s/L_s and orientation distribution θ/θ_0 of cracks in stereom. **d**, The distribution of parallel and transverse cracks in stereom.

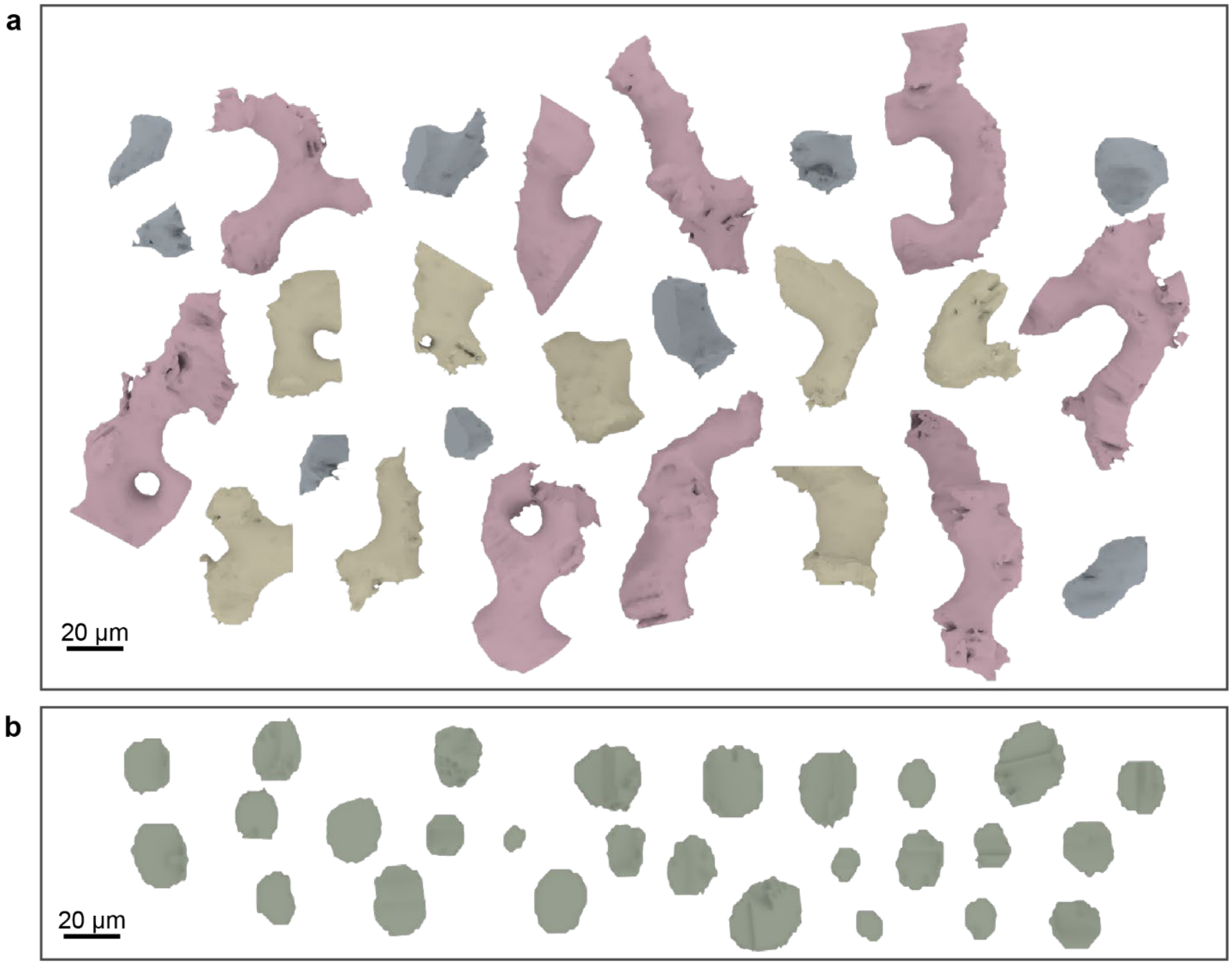

Supplementary Fig. 39. A qualitative comparison between the isolated fragments and throats in the stereom model in Fig. 38a. a,**b**, 3D renderings of (**a**) isolated fragments and (**b**) throats in the structure.

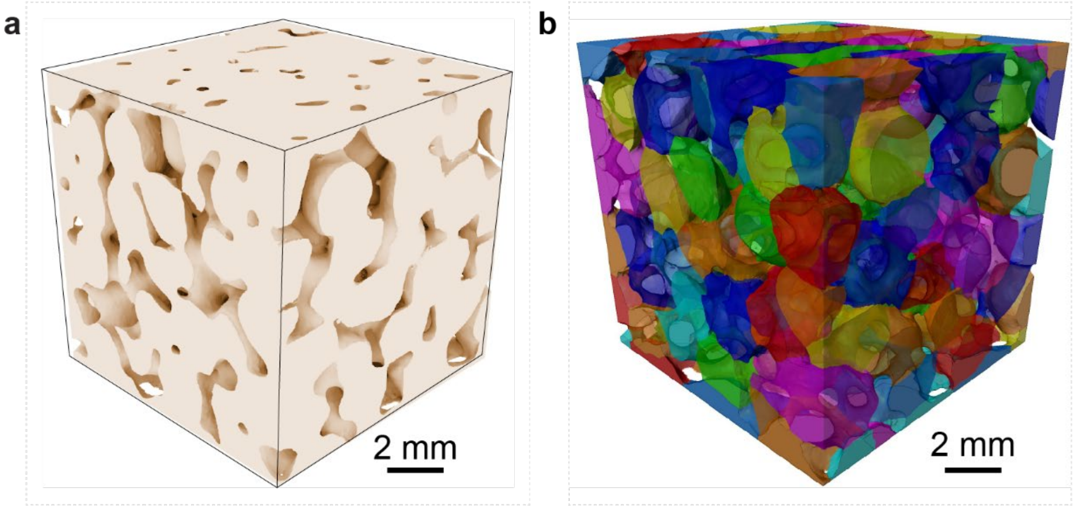

Supplementary Fig. 40. Void morphology in the reticulated foam. a, 3D rendering of the void in the reticulated foam. **b**, The void space can be further treated as an assembly of spherical void cells with diameter of ca. 2.9 mm.

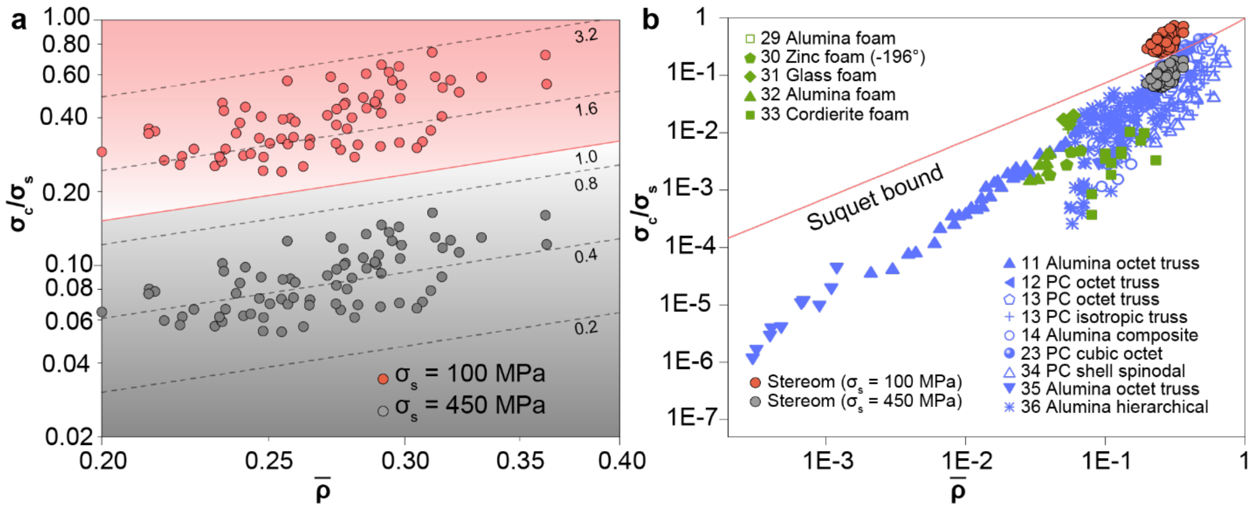

Supplementary Fig. 41. The Mechanical performance of stereom using different failure strength σ_s of solid struts ($\sigma_s = 100$ MPa and $\sigma_s = 450$ MPa) as the reference value. a, Distribution of relative strength of stereom, σ_c/σ_s, versus $\bar{\rho}$. **b,** The relative compressive strength of stereom in comparison to the traditionally[29-33] and additively[11-14,23,34-36] manufactured ceramic foams.

Locations	Close to center	Edge
**Relative density**	27%	37%
**Node density (solid, mm^{-3})**	40,000	60,000
**Node density (void, mm^{-3})**	40,000	35,000
**Predominant node type**	Three-branched node	Three-branched node
**Average nodal connectivity**	3.2	3.3
**Overlap between the distribution of d_s and d_t**	51.2%	57.7%

Supplementary Table 1. The structural properties comparison between the volumes with different relative densities.

Modulus	Stereom	Reticulated foam	3D printed octet truss
**Experiments**	4.3 ± 1.2 GPa	**N/A**	**N/A**
**FEM**	12.84 ± 2.08 GPa	8.12 ± 1.22 GPa	11.63 ± 0.04 GPa

Supplementary Table 2. Comparison of modulus measured from experiments and FEM among three ceramic foams

Strength	Stereom	Reticulated foam	3D printed octet truss
**Experiments**	40.40 ± 12.40 MPa	1.4 ± 0.3 MPa	2.2 ± 0.4 MPa
**FEM modeling**	23.95 ± 6.60 MPa	9.34 ± 2.41 MPa	14.69 ± 0.18 MPa

Supplementary Table 3. Comparison of compressive strength measured from experiments and FEM among three ceramic foams.

Species	Structure	Methods	Loading Planes	Mechanical Properties*		
				Modulus, E (GPa)	Hardness, H (GPa)	Strength, σ (GPa)
Atrina rigida	Prisms	Micro-pillar compression[8]	⊥{001}	$E_s = 34.4 \pm 5.3$		$\sigma_Y = 1.16 \pm 0.22$<br>$\sigma_f = 1.32 \pm 0.23$
		Nanoindentation[8]	⊥{001}	$E_r = 56.83 \pm 3.87$	2.83 ± 0.19	$\sigma_Y = 1.01 \pm 0.07$
		Nanoindentation[57]	⊥{001}	$E_{ps} = 74.9 \pm 0.7$	3.47 ± 0.21	$\sigma_Y = 1.24 \pm 0.08$
Placuna Placenta	Foliated laths	Micro-bending[9]	⊥{001}	$E_s = 36.24 \pm 14.41$		$\sigma_f = 0.45 \pm 0.14$
		Nanoindentation[58]	⊥{108}	$E_r = 71.1 \pm 4.2$	3.5 ± 0.3	$\sigma_Y = 1.25 \pm 0.11$
Heterocentrotus mamillatus	Growth ring	Nanoindentation[59]	⊥{001}	$E_r = 61.9 \pm 2.7$	3.7 ± 0.3	$\sigma_Y = 1.32 \pm 0.11$
	Medulla	Nanoindentation[59]	⊥{001}	$E_r = 39.8 \pm 6.1$	3.1 ± 0.4	$\sigma_Y = 1.11 \pm 0.14$
	Septa	Nanoindentation[59]	⊥{001}	$E_r = 50.5 \pm 3.0$	3.3 ± 0.3	$\sigma_Y = 1.18 \pm 0.11$
	Septa	Nanoindentation**	⊥{001}	$E_r = 79.31 \pm 3.61$	4.21 ± 0.28	$\sigma_Y = 1.50 \pm 0.10$
	Epithelium	Nanoindentation**	//{001}	$E_r = 88.87 \pm 2.34$	4.18 ± 0.20	$\sigma_Y = 1.49 \pm 0.07$
Phyllacanthus imperialis	Medulla	Nanoindentation[59]	⊥{001}	$E_r = 28.9 \pm 5.8$	2.8 ± 0.8	$\sigma_Y = 1.00 \pm 0.25$
	Septa	Nanoindentation[59]	⊥{001}	$E_r = 48.4 \pm 4.2$	4.0 ± 0.3	$\sigma_Y = 1.43 \pm 0.11$
Prinocidaris baculosa	Epithelium	Nanoindentation[59]	⊥{001}	$E_r = 71.7 \pm 3.8$	3.5 ± 0.2	$\sigma_Y = 1.25 \pm 0.07$
	Medulla	Nanoindentation[59]	⊥{001}	$E_r = 48.5 \pm 5.7$	3.5 ± 0.5	$\sigma_Y = 1.25 \pm 0.18$
	Septa	Nanoindentation[59]	⊥{001}	$E_r = 52.8 \pm 7.8$	3.7 ± 0.5	$\sigma_Y = 1.32 \pm 0.18$
Paracentrotus lividus	Central stereom	Nanoindentation[60]	⊥{001}	$E_r = 32.20 \pm 3.26$	1.76 ± 0.70	$\sigma_Y = 0.63 \pm 0.25$
	Septa	Nanoindentation[60]	⊥{001}	$E_r = 58.57 \pm 3.76$	3.84 ± 0.25	$\sigma_Y = 1.37 \pm 0.09$
Geological Calcite	N/A	Nanoindentation[57]	⊥{001}	$E_{ps} = 76.9 \pm 3.1$	2.30 ± 0.14	$\sigma_Y = 0.82 \pm 0.05$
		Nanoindentation[8]	⊥{001}	$E_r = 61.59 \pm 3.65$	1.81 ± 0.11	$\sigma_Y = 0.65 \pm 0.04$
		Nanoindentation[57]	⊥{104}	$E_{ps} = 88.1 \pm 1.7$	2.54 ± 0.07	$\sigma_Y = 0.91 \pm 0.03$
		Nanoindentation[58]	⊥{108}	$E_r = 73.42 \pm 1.74$	2.34 ± 0.10	$\sigma_Y = 0.84 \pm 0.04$
		Micro-pillar compression[8]	⊥{001}	$E_s = 47.9 \pm 5.5$		$\sigma_Y = 0.96 \pm 0.37$

Supplementary Table 4. Intrinsic mechanical properties of biogenic and geological calcite

* Modulus: E_s is the Young's modulus of the sample, E_r is the reduced modulus from indentation methods, E_{ps} is the plane strain modulus, and H is the hardness. $\frac{1}{E_r} = \frac{1-v_s^2}{E_s} + \frac{1-v_t^2}{E_t}$, and $E_{ps} = \frac{1-v_s^2}{E_s}$, where E_s and v_s are the Young's modulus and Poisson's ratio for the sample, and E_t and v_t are the corresponding properties for the indenter tip.

*Strength: σ_Y is the yielding strength, and σ_f is the ultimate failure strength. The gray boxes indicated yielding strength estimated from indentation hardness.
** The hardness and modulus of the stereom (septa and epithelium) were measured using nanoindentations in this paper (Methods).

Ceramic foams	Solid	Density ρ (g cm^{-3})	Solid density ρ_s (g cm^{-3})	Relative density $\bar{\rho}$	Failure strength σ_s (MPa)	Compressive strength σ (MPa)	Relative strength σ/σ_s	Diameter/layer thickness
**foam**[29]	Alumina	0.9	3.90	0.23	210	0.7	0.0033	/
**foam**[30]	Zinc (-196°)	0.29	7.14	0.04	/	/	0.00181	/
**Foam**[31]	Soda lime silica	0.13-0.15	2.55	0.05-0.06	/	0.96-1.91	0.014-0.02	/
**Foam**[32]	Alumina	0.11-0.15	3.95	0.029-0.038	/	/	0.0014-0.0043	/
**foam**[33]	Cordierite	0.23-0.52	2.91	0.08-0.18	65 ± 5[61]	0.10-1.87	0.0004-0.01	mm
**Shell spinodal**[34]	Glassy carbon	0.52-2.09	2.9	0.18-0.72	3400[62]	23.7-884	0.007-0.26	0.17-0.3 μm
**Octet truss**[11]	Alumina	0.006-0.12	2.9	0.002-0.04	2000	0.07-9	0.000035-0.0045	5 nm - 30 nm
**Octet truss**[35]	Alumina	0.00087-0.35	2.9	0.003-0.12	2200 (hollow); 1600 (solids)	0.003-7.36	0.0000012-0.0046	100nm - 2 mm
**filament with foams**[36]	Alumina	0.16-1.4	2.9	0.056-0.485		2	0.022-0.12	5 μm - 25 μm
**Octet truss**[12]	Glassy carbon	0.20-0.39	1.5	0.13-0.26	1400-3400	55 - 292	0.039-0.086	970–2020 nm
**Cubic octet**[23]	Glassy carbon	0.38-0.86	1.5	0.25-0.57	2200-2700	174-1000	0.07-0.43	150-260 nm
**Octet truss**[13]	Glassy carbon	0.225-0.6	1.4	0.24-0.68	1900	210-1730	0.01-0.09	261-679 nm
**Isotropic truss**[13]	Glassy carbon	0.72-1.08	1.4	0. 28-0.72	1900	140-1900	0.009-0.27	261-679 nm
**Octet truss**[14]	Alumina composites	0.166-0.421	/	0.12-0.19	3900 (10-50 nm); 1180 (100-200 nm)	6-33.2	0.0015-0.028	10-200 nm
**Global diagonal bracings**[14]	Alumina composites	0.127-0.322	/	0.094-0.146	3900 (10-50 nm); 1180 (100-200 nm)	4.5-26.9	0.0012-0.0028	10-200 nm
**Ortho-tropic construction**[14]	Alumina composites	0.117-0.223	/	0.073-0.101	3900 (10-50 nm); 1180 (100-200 nm)	3.6-16.5	0.0009-0.014	50-200 nm
**Hexagonal truss**[14]	Alumina composites	0.239-0.434	/	0.153-0.204	3900 (10-50 nm); 1180 (100-200 nm)	11-54.3	0.0028-0.046	50-200 nm
**Shape-optimized honeycomb**[14]	Alumina composites	0.463-0.863	/	0.34-0.452	3900 (10-50 nm); 1180 (100-200 nm)	109.6-273.6	0.028-0.070	10-200 nm

Supplementary Table 5. Mechanical properties of synthetic ceramic foams.

Supplementary References

1. Albéric, M. *et al.* Interplay between Calcite, Amorphous Calcium Carbonate, and Intracrystalline Organics in Sea Urchin Skeletal Elements. *Cryst. Growth Des.* **18**, 2189–2201 (2018).
2. Khan, N., Dollimore, D., Alexander, K. & Wilburn, F. W. The origin of the exothermic peak in the thermal decomposition of basic magnesium carbonate. *Thermochim. Acta* **367–368**, 321–333 (2001).
3. Wolf, S. L. P., Jähme, K. & Gebauer, D. Synergy of Mg2+ and poly (aspartic acid) in additive-controlled calcium carbonate precipitation. *CrystEngComm* **17**, 6857–6862 (2015).
4. Yang, T., Wu, Z., Chen, H., Zhu, Y. & Li, L. Quantitative 3D structural analysis of the cellular microstructure of sea urchin spines (I): Methodology. *Acta Biomater.* **107**, 204–217 (2020).
5. Reznikov, N. *et al.* Inter-trabecular angle: A parameter of trabecular bone architecture in the human proximal femur that reveals underlying topological motifs. *Acta Biomater.* **44**, 65–72 (2016).
6. Cheng, Y. T. & Cheng, C. M. Scaling approach to conical indentation in elastic-plastic solids with work hardening. *J. Appl. Phys.* **84**, 1284–1291 (1998).
7. Han, L., Wang, L., Song, J., Boyce, M. C. & Ortiz, C. Direct quantification of the mechanical anisotropy and fracture of an individual exoskeleton layer via uniaxial compression of micropillars. *Nano Lett.* **11**, 3868–3874 (2011).
8. Deng, Z. *et al.* Strategies for simultaneous strengthening and toughening via nanoscopic intracrystalline defects in a biogenic ceramic. *Nat. Commun.* **11**, 1–11 (2020).
9. Deng, Z. & Li, L. Intrinsic Mechanical Properties of Individual Biogenic Mineral Units in Biomineralized Skeletons. *ACS Biomater. Sci. Eng.* (2021).
10. Suquet, P. M. Overall potentials and extremal surfaces of power law or ideally plastic composites. *J. Mech. Phys. Solids* **41**, 981–1002 (1993).
11. Meza, L. R., Das, S. & Greer, J. R. Strong, lightweight, and recoverable three-dimensional ceramic nanolattices. *Science* **345**, 1322–6 (2014).
12. Bauer, J., Schroer, A., Schwaiger, R. & Kraft, O. Approaching theoretical strength in glassy carbon nanolattices. *Nat. Mater* **15**, 438–443 (2016).
13. Zhang, X., Vyatskikh, A., Gao, H., Greer, J. R. & Li, X. Lightweight, flaw-tolerant, and ultrastrong nanoarchitected carbon. *Proc. Natl. Acad. Sci. U. S. A.* **116**, 6665–6672 (2019).
14. Bauer, J., Hengsbach, S., Tesari, I., Schwaiger, R. & Kraft, O. High-strength cellular ceramic composites with 3D microarchitecture. *Proc. Natl. Acad. Sci.* **111**, 2453–2458 (2014).
15. Deshpande, V. S., Ashby, M. F. & Fleck, N. A. Foam topology: Bending versus stretching dominated architectures. *Acta Mater.* **49**, 1035–1040 (2001).
16. Bauer, J., Meza, L. R., Schaedler, T. A., Schwaiger, R., Zheng, X. & Valdevit, L. Nanolattices: An Emerging Class of Mechanical Metamaterials. *Adv. Mater.* **29**, 1–26 (2017).
17. Deshpande, V. S., Fleck, N. A. & Ashby, M. F. Effective properties of the octet-truss lattice material. *J. Mech. Phys. Solids* **49**, 1747–1769 (2001).
18. Messner, M. C. Optimal lattice-structured materials. *J. Mech. Phys. Solids* **96**, 162–183 (2016).
19. Dong, L., Deshpande, V. & Wadley, H. Mechanical response of Ti-6Al-4V octet-truss lattice structures. *Int. J. Solids Struct.* **60-61**, 107–124 (2015).
20. Pasini, D. & Guest, J. K. Imperfect architected materials : Mechanics and topology optimization. *MRS Bull.* **44**, 766–772 (2019).
21. Tancogne-dejean, T., Diamantopoulou, M., Gorji, M. B., Bonatti, C. & Mohr, D. 3D Plate-Lattices : An Emerging Class of Low-Density Metamaterial Exhibiting Optimal Isotropic Stiffness. *Adv. Mater.* **30**, 1803334.
22. Tancogne-Dejean, T. & Mohr, D. Elastically-isotropic truss lattice materials of reduced plastic

anisotropy. *Int. J. Solids Struct.* **138**, 24–39 (2018).
23. Crook, C. *et al.* Plate-nanolattices at the theoretical limit of stiffness and strength. *Nat. Commun.* **11**, 1–12 (2020).
24. Griffith, A. A. The phenomena of rupture and flow in solids. *Philos. Trans. R. Soc. London. Ser. A* **221**, 163–198 (1921).
25. Lampman, S. R. & Steven R. Lampman. *ASM Handbook: Volume 19: Fatigue and Fracture*. (ASM International, 1997).
26. Tromans, D. & Meech, J. A. Fracture toughness and surface energies of covalent minerals: Theoretical estimates. *Miner. Eng.* **17**, 1–15 (2004).
27. Ryne, A., Bisschop, J. & Dysthe, D. K. Experimental investigation of surface energy and subcritical crack growth in calcite. *J. Geophys. Res. Solid Earth* **116**, 1–10 (2011).
28. Lin, C. C. Elasticity of calcite: Thermal evolution. *Phys. Chem. Miner.* **40**, 157–166 (2013).
29. Yamada, Y. *et al.* Compressive deformation behavior of Al_2O_3 foam. *Mater. Sci. Eng. A* **277**, 213–217 (2000).
30. Thornton, P. H. & Magee, C. L. Deformation characteristics of zinc foam. *Metall. Trans. A* **6**, 1801–1807 (1975).
31. Morgan, J. S., Wood, J. L. & Bradt, R. C. Cell size effects on the strength of foamed glass. *Mater. Sci. Eng.* **47**, 37–42 (1981).
32. Brezny, R. & Green, D. J. The effect of cell size on the mechanical behavior of cellular materials. *Acta Metall. Mater.* **38**, 2517–2526 (1990).
33. Oliveira, F. A. C., Dias, S., Vaz, M. F. & Fernandes, J. C. Behaviour of open-cell cordierite foams under compression. *J. Eur. Ceram. Soc.* **26**, 179–186 (2006).
34. Guell Izard, A., Bauer, J., Crook, C., Turlo, V. & Valdevit, L. Ultrahigh Energy Absorption Multifunctional Spinodal Nanoarchitectures. *Small* **15**, 1903834 (2019).
35. Zheng, X., Lee, H., Weisgraber, T. H., Shusteff, M., DeOtte, J., Duoss, E. B., Kuntz, J. D., Biener, M. M., Ge, Q., Jackson, J. A., Kucheyev, S. O., Fang, N. X. & Spadaccini, C. M. Ultralight, ultrastiff mechanical metamaterials. *Science* **344**, 1373–1377 (2014).
36. Minas, C., Carnelli, D., Tervoort, E. & Studart, A. R. 3D Printing of Emulsions and Foams into Hierarchical Porous Ceramics. *Adv. Mater.* **28**, 9993–9999 (2016).
37. Qiao, J. C., Xi, Z. P., Tang, H. P., Wang, J. Y. & Zhu, J. L. Compressive property and energy absorption of porous sintered fiber metals. *Mater. Trans.* **49**, 2919–2921 (2008).
38. Aldoshan, A. & Khanna, S. Effect of relative density on the dynamic compressive behavior of carbon nanotube reinforced aluminum foam. *Mater. Sci. Eng. A* **689**, 17–24 (2017).
39. Fan, S., Zhang, T., Yu, K., Fang, H., Xiong, H., Dai, Y., Ma, J., Jiang, D. & Zhu, H. Compressive properties and energy absorption characteristics of open-cell nickel foams. *Trans. Nonferrous Met. Soc. China* **27**, 117–124 (2017).
40. Choy, S. Y., Sun, C. N., Leong, K. F. & Wei, J. Compressive properties of functionally graded lattice structures manufactured by selective laser melting. *Mater. Des.* **131**, 112–120 (2017).
41. Xie, B., Fan, Y. Z., Mu, T. Z. & Deng, B. Fabrication and energy absorption properties of titanium foam with CaCl2 as a space holder. *Mater. Sci. Eng. A* **708**, 419–423 (2017).
42. Stanev, L., Drenchev, B. & Yotov, A. Compressive Properties and Energy Absorption Behaviour of AlSi10Mg Open-Cell Foam. *J. Mater. Sci. Technol.* **22**, 44–53 (2014).
43. Maskery, I., Aboulkhair, N. T., Aremu, A. O., Tuck, C. J. & Ashcroft, I. A. Compressive failure modes and energy absorption in additively manufactured double gyroid lattices. *Addit. Manuf.* **16**, 24–29 (2017).
44. Yuan, S., Chua, C. K. & Zhou, K. 3D-Printed Mechanical Metamaterials with High Energy Absorption. *Adv. Mater. Technol.* **4**, 1800419 (2019).
45. Alvandi-Tabrizi, Y. & Rabiei, A. Use of Composite Metal Foam for Improving Absorption of Collision Forces. *Procedia Mater. Sci.* **4**, 377–382 (2014).
46. Gibson, L. J. The elastic and plastic behavior of cellular materials. (1981).

47. Walsh, J. B., Brace, W. F. & England, A. W. Effect of Porosity on Compressibility of Glass. *J. Am. Ceram. Soc.* **48**, 605–608 (1965).
48. Baxter, S. & Jones, T. T. Physical properties of foamed plastics and their dependence on structure. *PLASTICS & POLYMERS* **40**, 69 (1972).
49. Gent, A. N. & Thomas, A. G. The deformation of foamed elastic materials. *J. Appl. Polym. Sci.* **1**, 107–113 (1959).
50. Chan, R., & Nakamura, M. Mechanical properties of plastic foams: the dependence of yield stress and modulus on the structural variables of closed-cell and open-cell foams. *J. Cell. Plast.* **5(2),** 112-118 (1969).
51. Zheng, X. et al. Ultralight, ultrastiff mechanical metamaterials. *Science* **344**, 1373–1377 (2014).
52. Schaedler, T. A. et al. Ultralight metallic microlattices. *Science* **334**, 962–965 (2011).
53. Meza, L. R., Das, S. & Greer, J. R. Strong, lightweight, and recoverable three-dimensional ceramic nanolattices. *Science* **345**, 1322–6 (2014).
54. Worsley, M. A., Kucheyev, S. O., Satcher, J. H., Hamza, A. v. & Baumann, T. F. Mechanically robust and electrically conductive carbon nanotube foams. *Appl. Phys. Lett.* **94**, 1–4 (2009).
55. Moner-Girona, M., Roig, A., Molins, E., Martínez, E. & Esteve, J. Micromechanical properties of silica aerogels. *Appl. Phys. Lett.* **75**, 653–655 (1999).
56. Tillotson, T. M. & Hrubesh, L. W. Transparent ultralow-density silica aerogels prepared by a two-step sol-gel process. *J Non Cryst Solids* **145**, 44–50 (1992).
57. Kunitake, M. E., Mangano, L. M., Peloquin, J. M., Baker, S. P. & Estroff, L. A. Evaluation of strengthening mechanisms in calcite single crystals from mollusk shells. *Acta Biomater.* **9**, 5353–5359 (2013).
58. Li, L. & Ortiz, C. Pervasive nanoscale deformation twinning as a catalyst for efficient energy dissipation in a bioceramic armour. *Nat. Mater.* **13**, 501–507 (2014).
59. Presser, V., Gerlach, K., Vohrer, A., Nickel, K. G. & Dreher, W. F. Determination of the elastic modulus of highly porous samples by nanoindentation: A case study on sea urchin spines. *J. Mater. Sci.* **45**, 2408–2418 (2010).
60. Moureaux, C. *et al.* Structure, composition and mechanical relations to function in sea urchin spine. *J. Struct. Biol.* **170**, 41–49 (2010).
61. Costa Oliveira, F. A., Franco, J., Cruz Fernandes, J. & Dias, D. Newly developed cordierite-zircon composites. *Br. Ceram. Trans.* **101**, 14–21 (2002).
62. Albiez, A. & Schwaiger, R. Size Effect on the Strength and Deformation Behavior of Glassy Carbon Nanopillars. *MRS Adv.* **4**, 133–138 (2019).
